# Rapid Transition towards the Division of Labor via Evolution of Developmental Plasticity

**DOI:** 10.1371/journal.pcbi.1000805

**Published:** 2010-06-10

**Authors:** Sergey Gavrilets

**Affiliations:** Department of Ecology and Evolutionary Biology, Department of Mathematics, National Institute for Mathematical and Biological Synthesis, University of Tennessee, Knoxville, Tennessee, United States of America; University of Washington, United States of America

## Abstract

A crucial step in several major evolutionary transitions is the division of labor between components of the emerging higher-level evolutionary unit. Examples include the separation of germ and soma in simple multicellular organisms, appearance of multiple cell types and organs in more complex organisms, and emergence of casts in eusocial insects. How the division of labor was achieved in the face of selfishness of lower-level units is controversial. I present a simple mathematical model describing the evolutionary emergence of the division of labor via developmental plasticity starting with a colony of undifferentiated cells and ending with completely differentiated multicellular organisms. I explore how the plausibility and the dynamics of the division of labor depend on its fitness advantage, mutation rate, costs of developmental plasticity, and the colony size. The model shows that the transition to differentiated multicellularity, which has happened many times in the history of life, can be achieved relatively easily. My approach is expandable in a number of directions including the emergence of multiple cell types, complex organs, or casts of eusocial insects.

## Author Summary

Biological organisms are highly complex and are comprised of many different parts that function to ensure the survival and reproduction of the whole. How and why the complexity has increased in the course of evolution is a question of great scientific and philosophical significance. Biologists have identified a number of major transitions in the evolution of complexity including the origin of chromosomes, eukaryotes, sex, multicellular organisms, and social groups in insects. A crucial step in many of these transitions is the division of labor between components of the emerging higher-level evolutionary unit. How the division of labor was achieved in the face of selfishness of lower-level units is controversial. Here I study the emergence of differentiated cell colonies in which one part of the colony's cells (germ) specializes in reproduction and the other part of the colony's cells (soma) specializes in survival. Using a mathematical model I show that complete germ-soma differentiation can be achieved relatively easily and fast (with a million generations) via the evolution of developmental plasticity. My approach is expandable in a number of directions including the emergence of multiple cell types, complex organs, or casts of eusocial insects.

## Introduction

When biological units lose the ability to reproduce independently, and instead work together to reproduce collectively, a transition to a new level of organization occurs [1]–[4]. We refer to such collectives as organisms or individuals. During such transitions, the division of labor may evolve, where different low-level units specialize in different tasks to improve reproductive success of the organism. Examples include the separation of germ and soma in simple multicellular organisms, appearance of multiple cell types and organs in more complex organisms, and emergence of casts in eusocial insects [1], [2], [5]–[7].

Evolution of a higher level of organization can be viewed as a result of cooperation between specialized lower level units. However, cooperation is vulnerable to selfish cheating, and therefore explaining the emergence of the division of labor during such transitions is a major theoretical challenge [1], [2], [8], [9]. In the case of germ-soma differentiation, it has been suggested that fitness advantage of the division of labor can be sufficient to drive complete differentiation of cells and that selfish mutations and competion between cells do not disrupt the organism because cells are genetically identical (apart for somatic mutations) [2], [10]. Others, however, argue that these factors alone are insufficient to suppress cheating, and that additional mechanisms such as maternal control, early segregation of the germ line, mutual policing, and conflict mediation are necessary for the success of transitions [1], [11]–[14].

The complexity of the processes underlying major transitions in evolution and the accompanying division of labor accentuates the importance of mathematical modeling in augmenting and making more precise the conclusions based on generalization from data and empirical work. Earlier modeling work has focused on the fitness advantages of undifferentiated cell clusters, the benefits of within-colony specialization, the conditions for the spread of genetic modifiers decreasing cell defection or mutation rates, and the conditions for the evolutionary stability of terminally differentiated cells [4], [14]–[19].

Here I extend this work by examining developmental plasticity and considering the whole process of the emergence of a new level organization from initiation till completion. The scenario considered below focuses on two major genes regulated by two regulatory genes. The two major genes control cell's viability and fertility; due to fitness trade-offs these two functions cannot be optimized simultaneously. The regulatory genes react to an environmental stimulus (or stimuli) suppressing one or another major gene depending on the cell's position in the colony. The model identifies the conditions under which natural selection can drive the evolution of complete suppression of somatic function in one part of colony's cells (which become germ) and suppression of reproductive function in the other part of the colony's cells (which become soma). The outcome of these processes is the emergence of a new level of biological organization - a multicellular organism with complete germ-soma differentiation.

### Model

I consider a finite population of asexual haploid cells that form undifferentiated multicellular colonies by binary division. Mutation occur during cell divisions. Colonies surviving to the time of reproduction disintegrate; the released cells start new daughter-colonies. Each cell founding a colony goes through 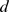 divisions so that the final colony size is 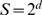 cells.

Each cell is characterized by viability 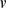 and fertility 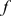. The former is a measure of the cell's contribution towards the survival of the colony it belongs to, e.g. via flagellar action [20], [21]. The latter is defined as the probability that the cell successfully starts a new colony. I assume the existence of two major genes with effects 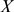 and 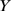 controlling cell fertility and viability, respectively (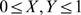). The direct effects of these genes increase the corresponding fitness components. To capture the fundamental trade-offs between cells division and locomotion capabilities [3], [4], [22], I postulate indirect negative effects of 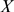 on viability and of 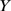 on fertility. Specifically, fertility and viability are defined using a simple multiplicative model:
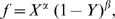


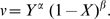
In the right-hand side of these equations, the first terms account for the direct effect of genes. Positive parameter 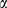 controls the shape of the relationships between direct genetic effect and the corresponding fitness component. The second terms specify the reduction of a fitness component due to the need to develop/maintain the other trait. Positive parameter 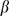 specifies the strength of fitness tradeoffs (which are completely absent if 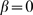). Because direct effects of genes are expected to be at least as strong as indirect effects, it is reasonable to assume that 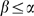.

The population of colonies is subject to density-dependent viability selection; all cells comprising surviving colonies can potentially form their own colonies in the next generation. Following previous work [4], [15], the viability 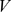 of each colony is defined as the average of viabilities of individual cells (i.e. 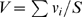). To describe viability selection at the colony level, I use a version of the Beverton-Holt model in which the probability that a colony survives to the time of reproduction depends on its viability 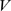 and the overall number of colonies 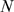 in the population:
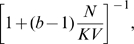
where 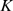 is the maximum carrying capacity of the population of colonies and parameter 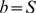 gives the number of “offspring” of each colony. In the deterministic version of the Beverton-Holt model (which represents a discrete-time analog of the logistic model [23]), the population size monotonically approaches the carrying capacity for any positive initial condition. The probability that a cell from a surviving colony does start a daughter colony is given by its fertility 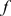. By the model's assumptions, the carrying capacity of a population of identical colonies is
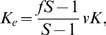
so that increasing cell viability 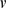 and/or fertility 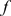 increases the number of colonies and cells maintained in the system; if the colony size 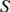 is very large, 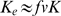. Note that in this model there is a conflict between individual level selection which favors larger values of 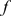 and colony level selection which favors larger values of 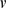. Both 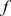 and 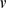 cannot be maximized simultaneously because of the trade-offs.

Mutation occurs during the process of cell division resulting in within- and between colony genetic variation. I assume each gene mutates with a small probability 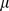 per cell division. Note that if a mutation does happen, the expected number of mutant cells per colony is 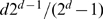 which is approximately 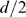 [24]. I assume that mutation changes the corresponding allelic effect (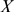 or 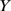) by a value chosen randomly and independently from a truncated Gaussian distribution with zero mean and a constant standard deviation 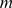 (with truncation at 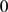 and 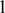). This is a version of the standard continuum-of-alleles model [25]. Note that a mutant cell in a colony will benefit if it has a higher value of 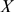 and/or smaller value of 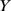 than other cells as this will increase the cell's fertility 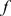. However such a cell will decrease the colony's viability 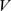.

Next I add a possibility for gene regulation. Molecular data suggest that in green algae *Volvox carteri*, which is a *bona fide* multicellular organism with a complete division of labor between two cell types [26], the germ-soma differentiation is controlled by three types of genes [20], [27], [28]. First, the *gls* genes cause asymmetric division resulting in a large number of small cells and a small number of large cells. Then the *regA* gene acts in small cells supressing their reproductive development, so that they become soma, and the *lag* gene acts in large cells supressing their somatic development, so that they become germ. Note that the expression of the *regA* gene has been shown to depend on environmental factors [29].

In the model, I postulate the existence of some dichotomy in the internal and/or external environment of the cells. For example, it can be asymmetry due to the differences in their size (large and small) or in their spatial position (e.g. inner and outer layer of the colony) leading to differences in some external stimuli (e.g. chemical or temperature). I call the two types of cells the proto-germ cells and the proto-soma cells. I assume that within each colony the proportion of the proto-germ cells is 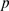 and that of the proto-soma cells is 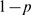. I further assume the existence of two differentially expressed regulatory genes with effects 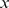 and 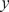, respectively (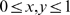). The first gene (analogous in action to the *lag* gene), is expressed in the proto-germ cells suppressing the effect of the “viability gene” from 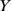 to 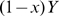. The second gene (analogous in action to the *regA* gene) is expressed in the proto-soma cells suppressing the effect of the “fertility gene” from 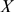 to 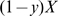. These two genes control the developmentally plastic response of the cell to the gradient in the internal and/or external environment. Note that in contrast to other modifiers studied in population genetic models [30]–[32], the two suppressor genes considered here have direct effect on fitness. This feature is common in theoretical models of phenotypic plasticity [33]–[35].

Since evolving gene suppression mechanisms and developmental plasticity is expected to involve fitness costs [36], [37], I assume that fertility of the proto-germ cells and viability of the proto-soma cells are reduced by factors 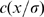 and 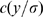, respectively. In numerical simulations I used Gaussian functions:
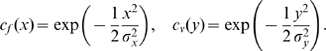
The costs grow as suppression becomes more efficient (i.e. with deviation of 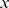 and 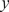 from zero); positive parameter 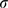 scales the costs of suppression (larger values correspond to smaller costs). Gene effects on reproductive and somatic function as well as fertility and viability of the proto-germ and proto-soma cells in the general model are shown in Table 1.

**Table 1:** Gene effects on reproductive and somatic function as well as fertility and viability of the proto-germ and proto-soma cells in the general model.

	Gene effects		Fitness components	
	reproductive function	somatic function	fertility 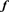	viability 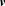
proto-germ	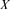	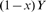	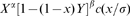	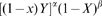
proto-soma	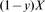	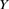	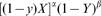	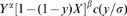

The initial population of cells have all 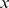 and 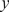 values set at 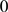 so that no gene suppression is present. I allow for mutation in the regulatory genes and describe its effect in a way analogous to that in the major loci. The complete germ-soma differentiation corresponds to 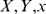 and 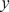 all evolving to 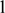 so that germ cells have maximum fertility but cannot survive on their own while soma cells have maximum viability but cannot reproduce.

## Results

**Figure 1:**
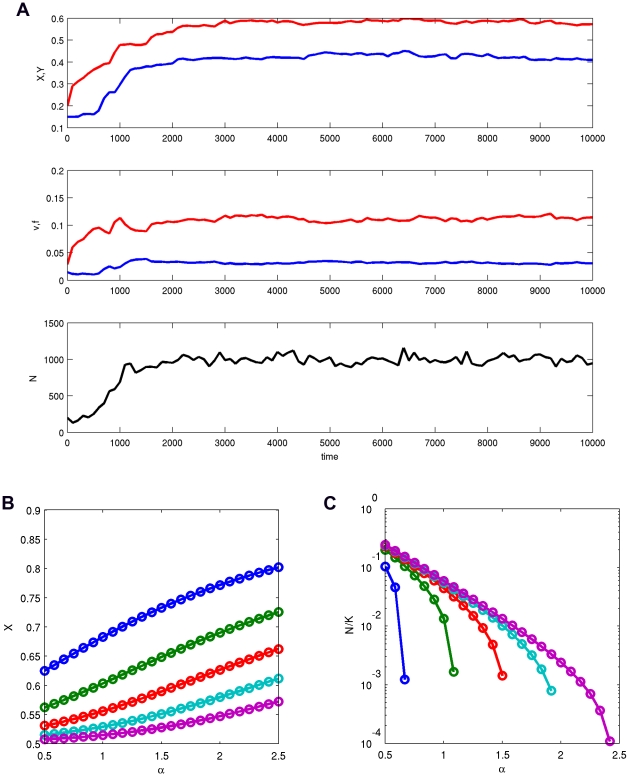
Evolution in major loci. (A) An example of the model's dynamics with 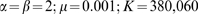. Shown are at top: the average values of 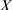 (red) and 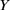 (blue), middle: the average fertility 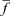 (red) and viability 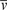 (blue), and bottom: the number of colonies 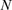 in the system. (B) The equilibrium values of 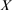 for different 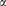 and 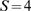 (blue),8, 16, 32 and 64 (pink). 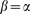, so that 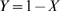. (C) The relative equilibrium population size 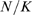 for the same values of parameters as in (b).

First I studied a variant of the general model in which gene regulation was absent (i.e., 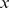 and 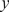 values were set to zero). I used a multidimensional invasion analysis [38]–[43] and stochastic individual-based numerical simulations (see **Methods** for details). Both methods show that in this model the major gene effects 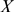 and 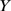 relatively rapidly evolve towards intermediate values so that both fitness components and the population size are relatively low (see Figure 1). The inability to increase fitness is a consequences of fitness trade-offs explicitly accounted for by the model.

Analytical approximations show that the equilibrium values of 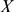 and 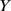 satisfy to inequalities 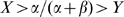. As the strength of fitness tradeoffs 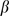 decreases to 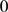, both 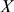 and 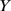 approach 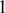. As the colony size 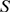 becomes larger, both equilibrium values converge to 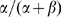. If 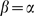, then at equilibrium 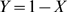 with 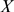 given by a solution of an algebraic equation 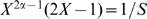. In general, analytical and numerical results show that increasing the strength of selection 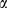, the strength of trade-offs 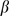, and decreasing the colony size 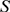 result in decreasing both fitness components and the population size.

**Figure 2:**
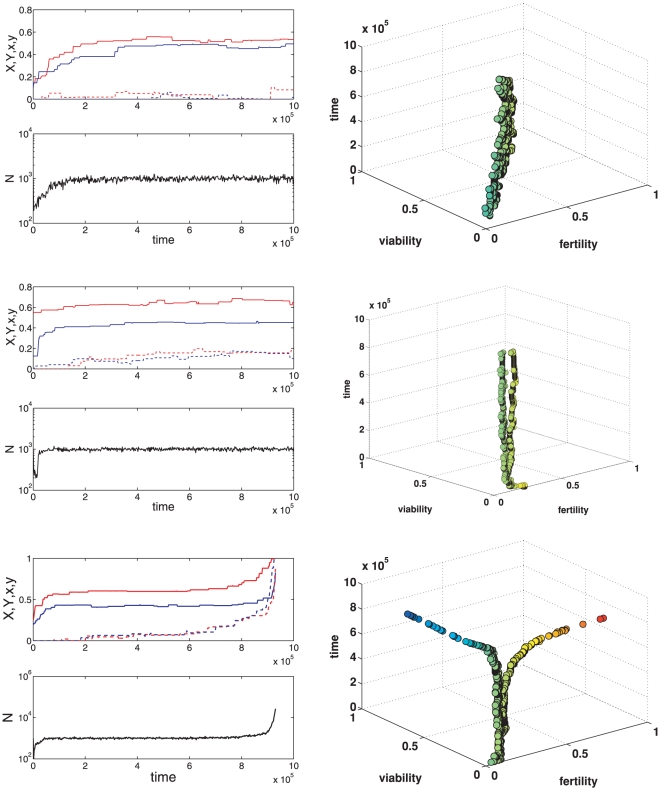
Examples of the model dynamics with 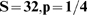. First column: the dynamics of the main (solid lines) and modifier (dashed lines) allelic effects and the population size. Second column: fertility and viability for pro-some and proto-germ cells; each cell in the population is represented by a circle. Data are saved every 2000 generations. First row: 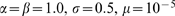. Second row: 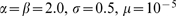. Third row: 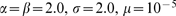.

To analyze the whole model I performed large-scale stochastic individual-based simulations that account for selection, mutation, and random genetic drift (see **Methods**). For each run, all individuals in the initial population were genetically identical with the major locus effects 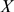 and 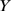 set to values chosen randomly and independently from a uniform distribution on 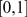 and the suppressor effects 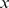 and 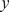 set to zero. The simulations show that the initial phase of evolution is typically driven by selection on the major loci whose effects evolve towards the optimum values predicted by our theory when developmental plasticity is absent (as in Figure 1). After that there are three dynamic possibilities. First, the population stays at a state in which developmental plasticity is absent (so that 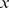 and 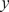 remain close to 0; Figure 2, first row). Second, some developmental plasticity evolves but the resulting degree of differentiation between proto-germ and proto-soma cells is intermediate (Figure 2, second row). Third, one observes the evolution of strong developmental plasticity and complete germ-soma differentiation (Figure 2, third row).

**Figure 3:**
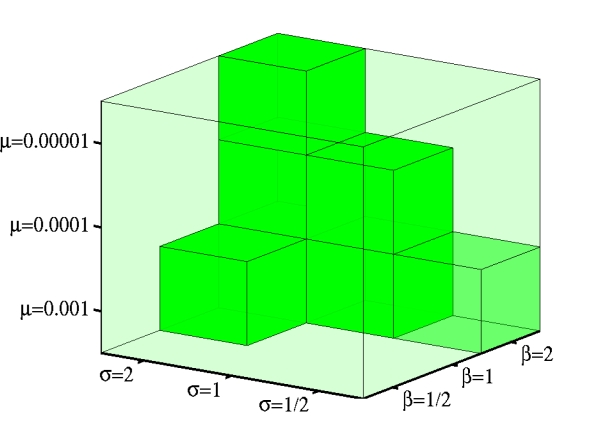
The areas of the 3-dimensional parameter space 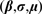 where complete germ-soma differentiation was observed (filled cubes). 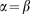
. For 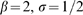, and 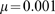 (lightly colored subcube), the major locus effects 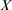 and 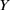 evolved very close to 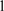 but the modifier effects 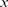 and 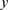 were around 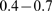.

The last outcome is observed when costs of developmental plasticity are small, mutation rates are high, and fitness trade-offs are strong (Figure 3). The effects of increasing costs of plasticity 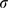 and mutation rate 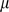 on the plausibility of differentiation are intuitive. Indeed, less constraints and more genetic variation typically means more adaptation. But why do fitness trade-offs have such a big effect? This happens because larger values of 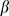 imply that fitness advantage of a highly differentiated state is larger. For example, for the parameter values used in the simulations the size of the equilibrium population of undifferentiated colonies is 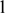 thousand. However, the size of the equilibrium population of completely differentiated colonies will be about 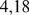, and 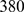 thousand for 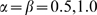 and 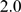, respectively. That is, the benefit of cell differentiation for the population size (and fitness) increases dramatically with 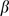. The results shown in Figures 2–3 as well as in **Supporting Information** (Text S1 and Figures S1, S2, S3, S4, S5, S6, and S7) are for 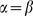. If 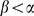, the conditions for complete differentiation are more strict. Neither the proportion of the proto-germ cells 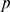 nor the colony size 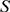 affect the results qualitatively.

**Figure 4:**
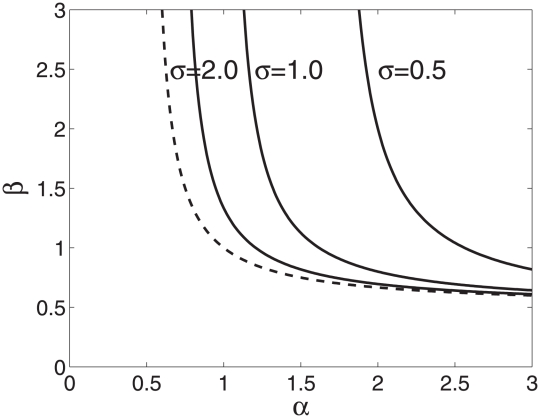
Conditions for local stability of an equilibrium with no gene supression (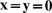) and optimum value of major locus effects (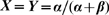) when the colony size is very large (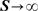) for 3 different values of 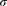 (shown on the graph). The equilibrium is stable for 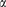 and 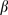 values on the left of the corresponding curve. The dashed curve corresponds to no costs of gene supression (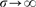).

Analytical approximations for the case when the colony size is very large (i.e. 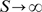) allow one to get some additional insights. In particular, one can find the conditions for stability of a population state with no gene regulation (i.e., 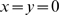) towards introduction of mutations with small positive values of 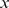 and 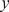. These conditions are illustrated in Figure 4 which shows that this equilibrium becomes unstable so that some gene suppression evolves if parameters 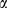 and 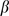 are sufficiently large and the cost of developmental plasticity is low (i.e. 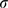 is not too small). Moreover, one can show that if fitness trade-offs are sufficiently strong (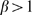) then the corresponding dynamic system has an equilibrium in which major effects have maximum possible values (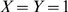) whereas the minor gene effects are 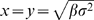. The later value is biologically feasible (so that 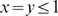), if fitness costs of plasticity are sufficiently high (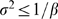). If 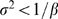, only partical gene suppression evolves. If the costs are relatively low (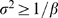), the analytical approximations suggest that complete gene suppression evolves (i.e., 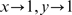). These results are well in line with numerical simulations described above.

## Discussion

The model introduced and analyzed here shows the emergence of complete germ-soma differentiation. This is achieved via the evolution of developmental plasticity resulting in the suppression of somatic function in one subset of the colony's cells and of reproductive function in the remaining cells of the colony. Differential suppression of gene expression is triggered by environmental factors during development. A necessary condition for this process is the existence of sufficiently strong trade-offs between somatic and reproductive functions significantly reducing fitness. Also necessary are sufficiently high mutation rates and sufficiently low costs of developmental plasticity. With parameter values used here, complete germ-soma differentiation can evolve within a million generations.

The model proposed here is simple and biologically realistic in capturing the major features of volvocine green algae biology [20], [26]–[28] that are relevant for the germ-soma differentiation. [The model does not account for the *gls* genes introducing asymmetry in size between proto-germ and proto-soma cells, but asymmetric division was a late, lineage-specific step in volvocine evolution [44].] The results presented clearly show that fitness advantages of the division of labor in the presense of strong genetic relatedness of cells in a colony are sufficient to drive the complete differentiation of cells [2], provided mutations that altruistically remove lineages from the germ line are expressed conditionally [10], [45]. Conditionally expressed genes allow the benefits of altruism to go to cells that possess, but do not express, the same allele [10].

In the model, cell differentiation and the division of labor are driven by individual selection maximizing the number of colony-producing offspring of a colony-producing cell. That is, the transition to individuality can be explained in terms of immediate selective advantage to individual replicators [2]. Note that mutant cells that “cheat” by having increased fertility within colonies will tend to lose in competition at the colony level after they develop their own colonies. Therefore, the conflict between individual and colony level selection is largely removed. The division of labor is achieved by using the variation in external and/or internal cell environment as a cue to separate the colony's cells by function and then enhance different functions using different subsets of cells.

The colony size 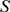 has no significant effect on the model dynamics. In contrast, in Volvox the degree of differentiation between germ- and soma-like cells does correlate with the colony size [26]: species with small colonies (8–32 cells) show no cell differentiation, in species with intermediate colonies (64–128 cells) incomplete germ-soma differentiation is observed, and differentiation is complete in species forming large colonies (500–5000 cells). However there is a number of biological factors not included in the model explicitly but acting in real cells and colonies which should result in a positive relationship between the colony size and the degree of differentiation. First, one can reasonably argue that a sufficiently large colony size is necessary for the existence of sufficiently strong gradients in the external environment to which the regulatory genes can react to. Second, increasing the colony size should result in some spatial heterogeneity between cells in their ability to perform different functions. For example, inner-layer cells are likely to be less important in contributing towards the colony motility than the outer-layer cells. Such heterogeneity should decrease the cost of loosing certain functions for some parts of the colony and make the evolution of cell differentiation easier. Third, the total number of cells performing a particular function in very small colonies may be too small to guarantee an appropriate level of performance especially if the probability of breakage per cell is not small.

A potentially important role for developmental plasticity in the evolution of differentiated multicellularity was emphasized earlier by Schlichting ([46]; see also [29]) but from a different perspective. Schlichting's argument was that cell differentiation started as a by-product of random environmental effects translated into new phenotypic forms via pre-existing reaction norms. Then later favorable phenotypic differentiation became canalized and stabilized via genetic assimilation process. In contrast, in the scenario considered here developmental plasticity is absent initially and emerges later as a direct result of selection.

Few additional points and connections are worth to be made. First, the model assumes the existence of undifferentiated multicellular colonies. Undifferentiated multicellularity has a number of advantages (e.g. size related) over single-celled organization and is expected to evolve relatively easily [3], [16], [18], [47], [48]. Second, empirical data show a strong positive relationship between the number of cells in an organism and a number of cell types [5], [49], [50]. The classical explanation of this pattern is that increasing the number of cells changes fitness landscape (e.g. due to physical constraints) in such a way that differentiation and specialization become necessary for optimizing the efficiency of organisms [5], [49], [51]. In our simple model, the fitness landscape is unaffected by the number of cells in the colony so the model in its current form cannot be used for addressing the question about the relationsips between the number of cells and cell types. Third, the model is also relevant to ongoing work and discussions on the importance and evolution of modularity, i.e. the separability of the design into units that perform independently, at least to a first approximation [52]–[54]. Although there is an emerging agreement that organisms have a modular organization, one of the major open questions is whether modules arise through the action of natural selection or because of biased mutational mechanisms [53]. In the model considered here, the modules (e.g. germ and soma) clearly emerge as a result of selection for reduced fitness trade-offs. Finally, I should mention some parallels between the model's structure and dynamics and the arguments on “groundplans” [55]–[57] according to which the patterns of labor division in complex organisms and societies are built upon simple changes in the regulation of conserved ancestral genes affecting reproductive physiology and behavior.

The model presented here is expandable in a number of directions including the emergence of multiple cell types, complex organs, or casts of eusocial insects. For example, the emergence of multiple cell types can be modeled by considering additional cell functions and introducing additional regulatory genes. The evolution of casts of eusocial insects can be explored by explicitly accounting for regulatory genes that react to the external stumuli (e.g, food level or pheromones) affected by the colony's composition. The majority of existing models of the division of labor in eusocial insects focus on individual worker flexibility in task performance [58], [59]. In contrast, the approach introduced here concentrates exclusively on genetically predetermined roles that do not change in time. Note that genetic variation present in some insect colonies (e.g. due to polyandry, [60]) will result in reduced genetic relatedness and, thus, is expected to make conditions for the evolution of the division of labor more strict.

The main result that complete cell differentiation evolves relatively easily and fast supports the view that the transition to differentiated multicellularity, which has happened at least two dozen times in the history of life, is in a sense actually a minor major transition [3], [8], [61], [62].

## Methods

### Fitness, carrying capacity, and invasion fitness

It is natural to define fitness as the expected number of offspring colonies in the next generation for a cell starting a colony. Then, for a cell characterized by viability 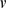 and fertility 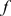, fitness is
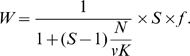
(1)In the model, the number of colonies of cells with viability 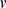 and fertility 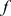 changes approximately according to
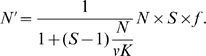
Therefore the number of colonies evolves towards the carrying capacity
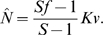
(2)Assuming that the ecological dynamics (i.e. changes in the population size) occur on the faster times scale than the evolutionary dynamics, the (invasion) fitness 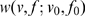 of a mutant cell 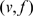 in a resident population 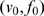 is given by eq.1 with 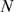 given by eq. 2 corresponding to the resident population. Simplifying,
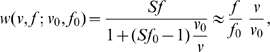
where the approximation is good only if 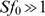. Note that the derivative of the invasion fitness function (with respect to a particular independent variable) evaluated at the resident population values can be written as
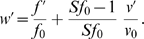



### Evolution of major loci

With only major gene effects 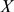 and 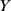 evolving (and minor gene effects 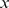 and 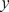 set at zero), the corresponding invasion fitness gradients are
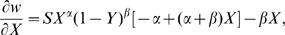


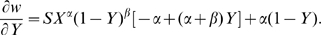
At an equilibrium (i.e., at a singularity), 
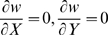
. From the first equation, it follows that at equilibrium 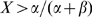 and that 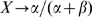 as 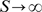. From the second equation, it follows that at equilibrium 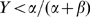 and that 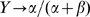 as 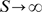. Eliminating the term 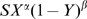 from the equalities 
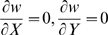
, one finds that at equilibrium
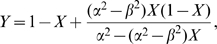
which is greater than 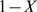 for 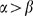. If 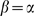, then 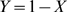 with 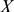 given by a solution of equation 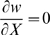 which simplifies to
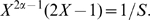
Note that 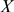 stays above 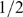 decreasing to it only asymptotically as 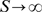. If 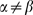, the equilibrium values of 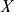 and 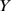 can still be found numerically from the above system of equations.

### Evolution of minor loci

In the general model, fertility and viability of a monomorphic colony can be written as
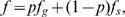


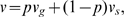
where 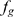 and 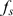 are fertilities and 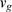 and 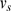 are viabilities of the proto-germ and proto-soma cells (as defined in Table 1), and 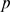 is the proportion of proto-germ cells in the colony.

Multidimensional invasion analysis requires one to consider four invasion fitness gradients: 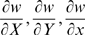 and 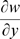. Some analytical progress can be achieved if the colony size is very large (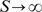). Under this condition, both major locus effects evolve to 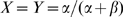 (see the previous subsection). Then we can study the stability of the equilibrium with no gene regulation (i.e., with minor locus effect 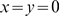) to introduction of mutants with small 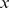 and 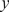. The corresponding invasion fitness gradients are approximated by equations linear in 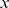 and 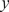:
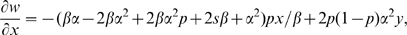


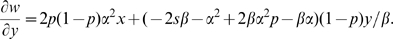
where 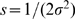. Assuming equal genetic variation maintained in both genes, standard linear stability analysis shows that an equilibrium with no gene regulation is locally unstable if
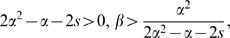
and is stable otherwise. Figure 4 in the main text illustrates this result.

By considering the four invasion fitness gradients simultaneously (while still assuming that 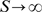), one can show that if 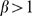, there exists a singular point at which 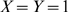 and 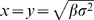. This suggests that if costs of developmental plasticity are not too big (i.e., if 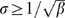, then maximum possible gene suppression evolves (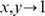). Overwise, the minor gene effects stay at intermediate values (i.e., between 0 and 1). Note that with 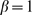 and 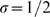, the predicted values of 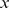 and 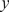 are 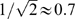 which is very close to the values observed in numerical simulations with 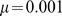 (see the legend of Figure 4).

Unfortunately, similar simple approach cannot be used for an arbitrary 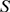 because the equilibrium values of the major locus effects cannot be found explicitly.

### Numerical results

In numerical simulations I used all possible combinations of the following parameters: fitness trade-off coefficients 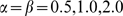, costs of developmental plasticity 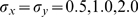; mutation rates 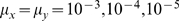; number of divisions 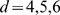 (so that the colony size was 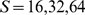); proportion of the proto-germ cells 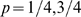. Mutational standard deviation was set to 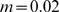. The maximum carrying capacity 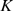 was chosen so that the population with no developmental plasticity (i.e. with 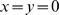) evolved to a state at which the number of colonies was close to 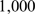. For example, with 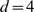, 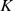 was set to 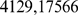, and 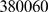 for 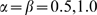 and 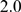, respectively. First, I run the model 3 times for each parameter combination each for 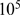 generations. Then for parameter values resulting in no differentiation, I did one additional run for 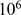 generations.

A gallery of numerical results can be viewed in Supporting Information (Text S1 and Figures S1, S2, S3, S4, S5, S6, S7, and S8).

## Supporting Information

Text S1 Supporting information details.
(0.01 MB DOC)

Figure S1 Numerical results for S = 16 and p = 1/4.
(1.57 MB PDF)

Figure S2 Numerical results for S = 16 and p = 3/4.
(1.57 MB PDF)

Figure S3 Numerical results for S = 32 and p = 1/4.
(1.61 MB PDF)

Figure S4 Numerical results for S = 32 and p = 3/4.
(1.60 MB PDF)

Figure S5 Numerical results for S = 64 and p = 1/4.
(1.62 MB PDF)

Figure S6 Numerical results for S = 64 and p = 3/4.
(1.63 MB PDF)

Figure S7 Numerical results for 1M runs.
(0.54 MB PDF)
