# Supplementary material for: Rapid Transition towards the Division of Labor via Evolution of Developmental Plasticity
Source: PLoS Comput Biol. 2010 Jun 10;6(6):e1000805. doi: 10.1371/journal.pcbi.1000805 (PMC2883585; doi:10.1371/journal.pcbi.1000805)
Supplement: Text S1 — Supporting information details. (0.01 MB DOC) [file pcbi.1000805.s001.doc]

# Supplementary online material

# Gallery of results

Each of Figures S1-S6 shows 3 runs for 100,000 generations for all possible combinations of
parameters alpha=beta=[0.5 1.0 2.0], sigma=[0.5 1 2.0], and mu=[0.001 0.0001 0.00001]. Numerical

values of S and p are specified in the figure title; m=0.02 always. Different runs are shown on the same row. In each figure, the top half of graphs show the average allelic effects X,Y,x and y, the average fertility f and viability v and the population size N. The bottom half of graphs show the dynamics of individual viabilities and fertilities (as in the right column of Figure 2 of the main text).

Figure S7 shows 1 run for 1,000,000 generations for the same combinations of alpha, sigma and mu but with S=32 and p=1/4 only.

In all figures the parameter values are marked on top of the pages (S and p) and below the corresponding graph (alpha,beta and mu).
