# Supplementary material for: Rapid Transition towards the Division of Labor via Evolution of Developmental Plasticity
Source: PLoS Comput Biol. 2010 Jun 10;6(6):e1000805. doi: 10.1371/journal.pcbi.1000805 (PMC2883585; doi:10.1371/journal.pcbi.1000805)
Supplement: Figure S3 — Numerical results for S = 32 and p = 1/4. (1.61 MB PDF) [file pcbi.1000805.s004.pdf]

S=32.p=0.25

Number of images: 162
Created on: Monday 29 March 2010

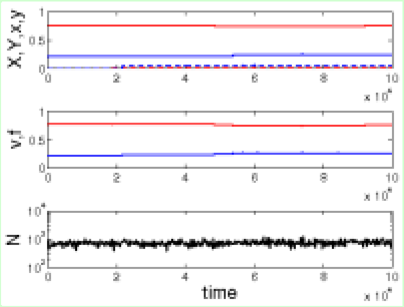

a=0.5.b=0.50.s=0.5.mu=0.00001.1.eps

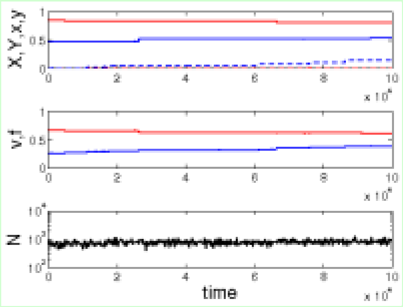

a=0.5.b=0.50.s=0.5.mu=0.00001.2.eps

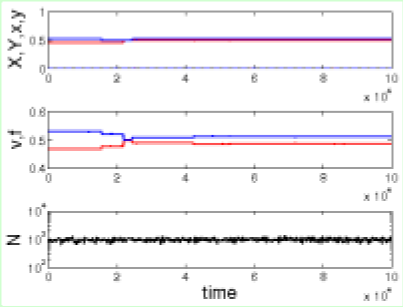

a=0.5.b=0.50.s=0.5.mu=0.00001.3.eps

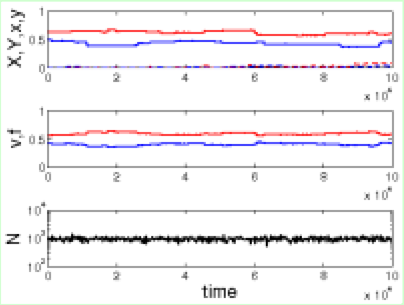

a=0.5.b=0.50.s=0.5.mu=0.00010.1.eps

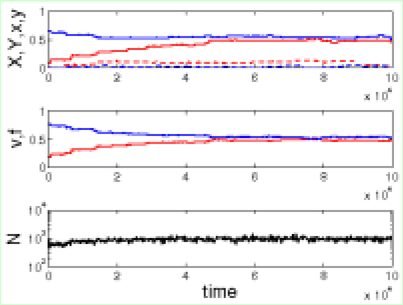

a=0.5.b=0.50.s=0.5.mu=0.00010.2.eps

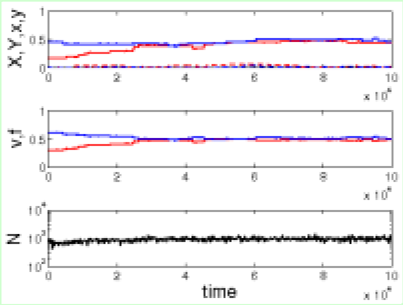

a=0.5.b=0.50.s=0.5.mu=0.00010.3.eps

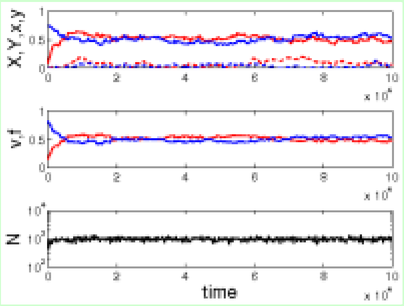

a=0.5.b=0.50.s=0.5.mu=0.00100.1.eps

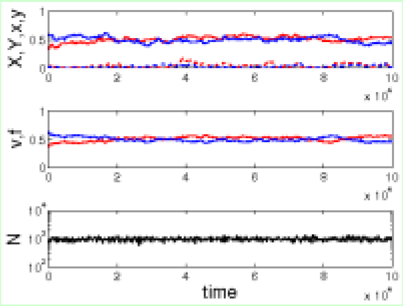

a=0.5.b=0.50.s=0.5.mu=0.00100.2.eps

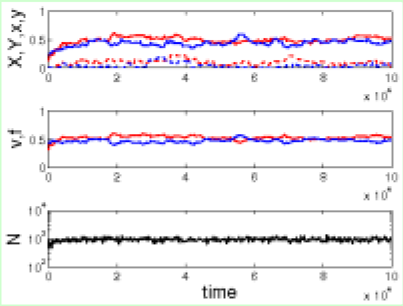

a=0.5.b=0.50.s=0.5.mu=0.00100.3.eps

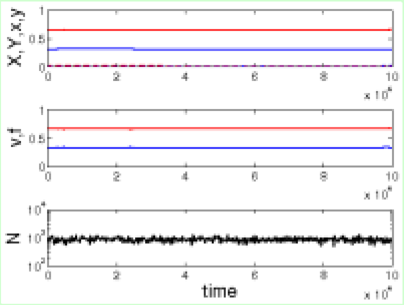

a=0.5.b=0.50.s=1.0.mu=0.00001.1.eps

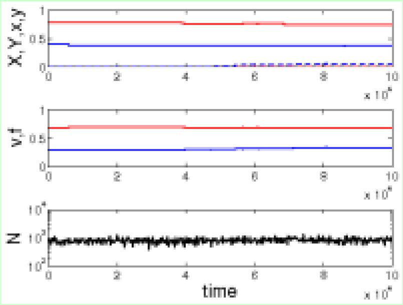

a=0.5.b=0.50.s=1.0.mu=0.00001.2.eps

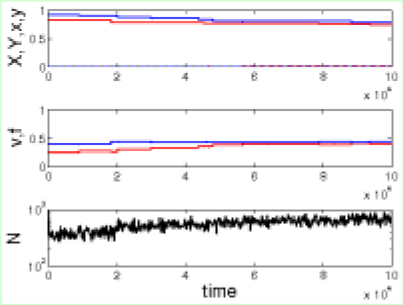

a=0.5.b=0.50.s=1.0.mu=0.00001.3.eps

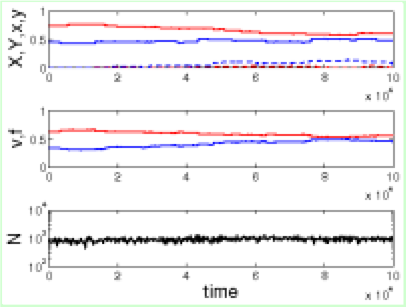

a=0.5.b=0.50.s=1.0.mu=0.00010.1.eps

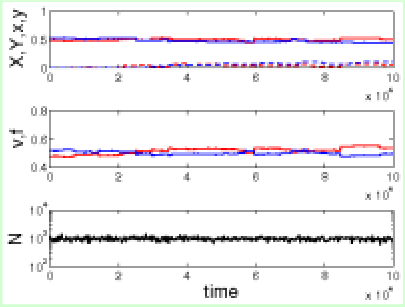

a=0.5.b=0.50.s=1.0.mu=0.00010.2.eps

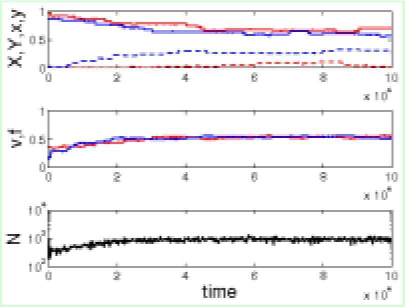

a=0.5.b=0.50.s=1.0.mu=0.00010.3.eps

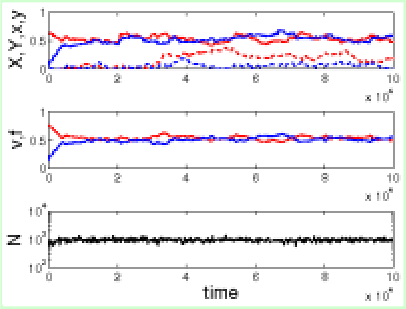

a=0.5.b=0.50.s=1.0.mu=0.00100.1.eps

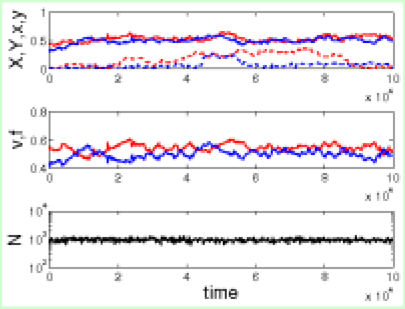

a=0.5.b=0.50.s=1.0.mu=0.00100.2.eps

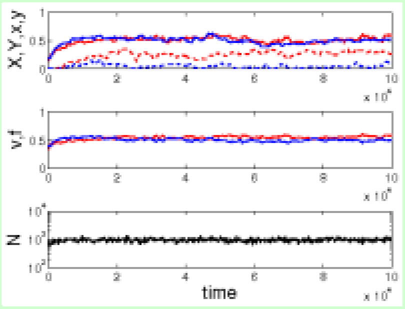

a=0.5.b=0.50.s=1.0.mu=0.00100.3.eps

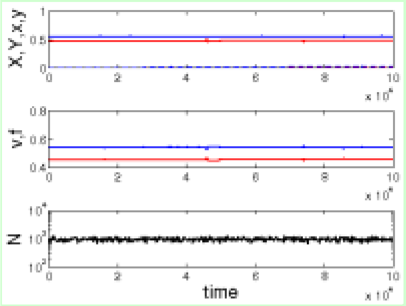

a=0.5.b=0.50.s=2.0.mu=0.00001.1.eps

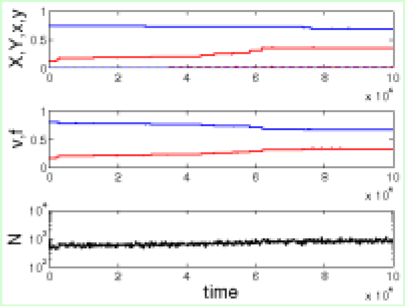

a=0.5.b=0.50.s=2.0.mu=0.00001.2.eps

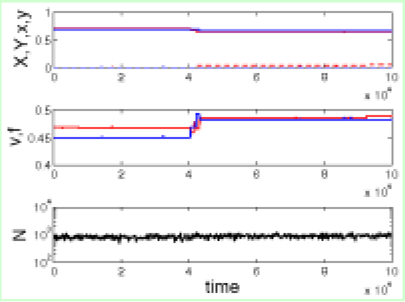

a=0.5.b=0.50.s=2.0.mu=0.00001.3.eps

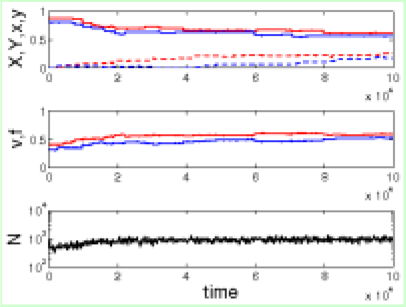

a=0.5.b=0.50.s=2.0.mu=0.00010.1.eps

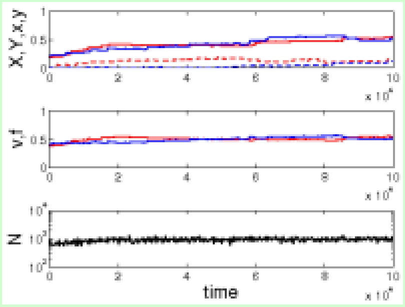

a=0.5.b=0.50.s=2.0.mu=0.00010.2.eps

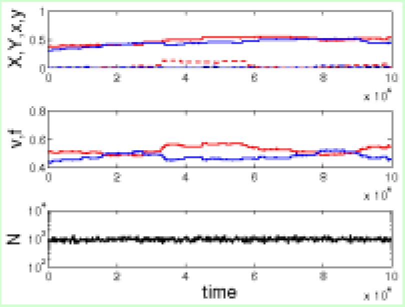

a=0.5.b=0.50.s=2.0.mu=0.00010.3.eps

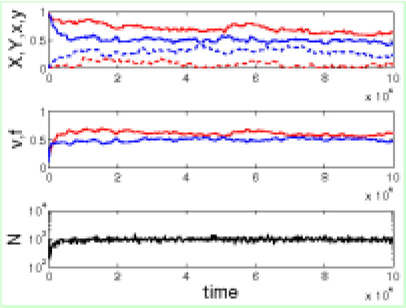

a=0.5.b=0.50.s=2.0.mu=0.00100.1.eps

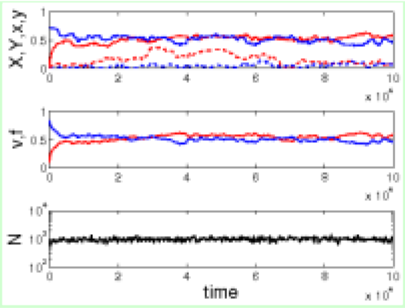

a=0.5.b=0.50.s=2.0.mu=0.00100.2.eps

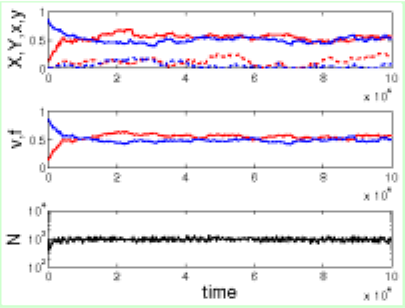

a=0.5.b=0.50.s=2.0.mu=0.00100.3.eps

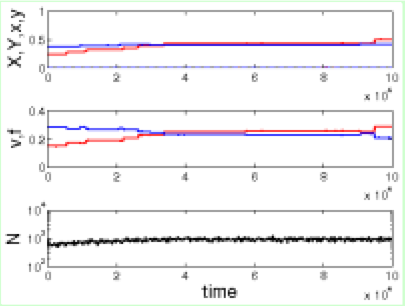

a=1.0.b=1.00.s=0.5.mu=0.00001.1.eps

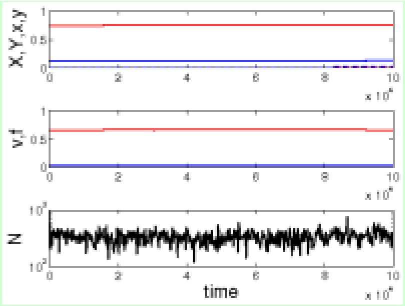

a=1.0.b=1.00.s=0.5.mu=0.00001.2.eps

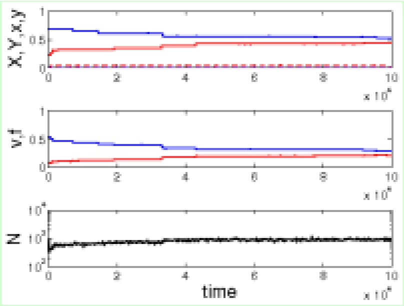

a=1.0.b=1.00.s=0.5.mu=0.00001.3.eps

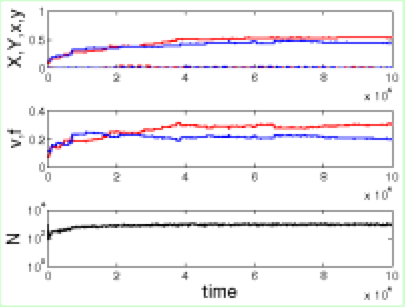

a=1.0.b=1.00.s=0.5.mu=0.00010.1.eps

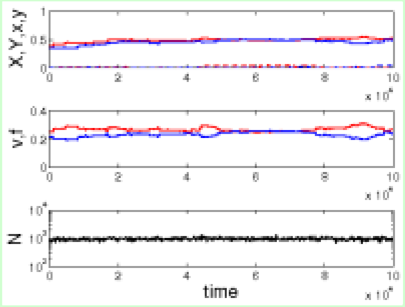

a=1.0.b=1.00.s=0.5.mu=0.00010.2.eps

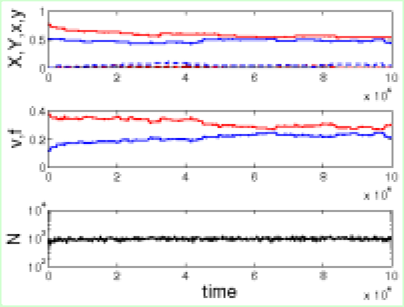

a=1.0.b=1.00.s=0.5.mu=0.00010.3.eps

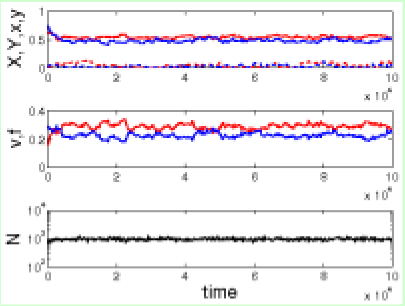

a=1.0.b=1.00.s=0.5.mu=0.00100.1.eps

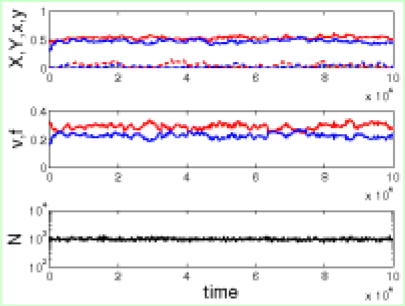

a=1.0.b=1.00.s=0.5.mu=0.00100.2.eps

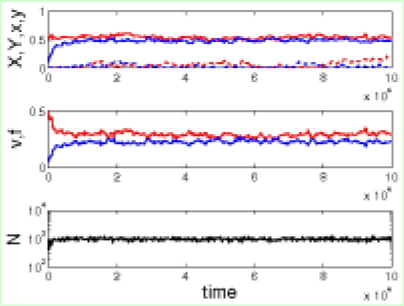

a=1.0.b=1.00.s=0.5.mu=0.00100.3.eps

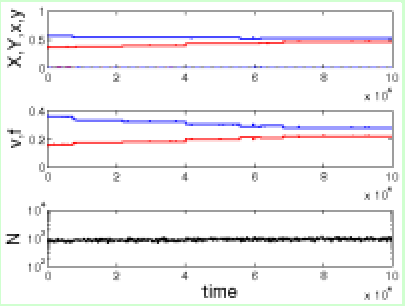

a=1.0.b=1.00.s=1.0.mu=0.00001.1.eps

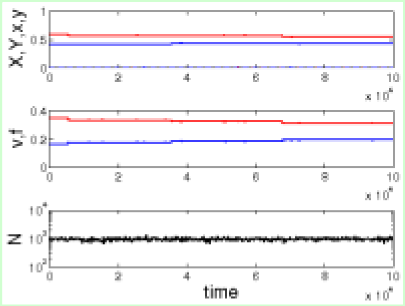

a=1.0.b=1.00.s=1.0.mu=0.00001.2.eps

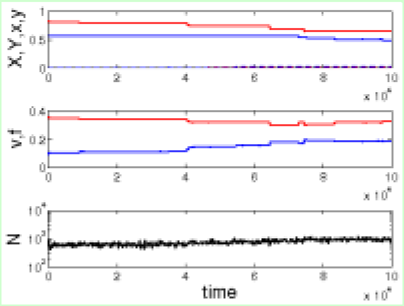

a=1.0.b=1.00.s=1.0.mu=0.00001.3.eps

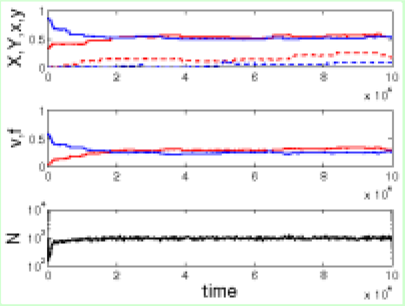

a=1.0.b=1.00.s=1.0.mu=0.00010.1.eps

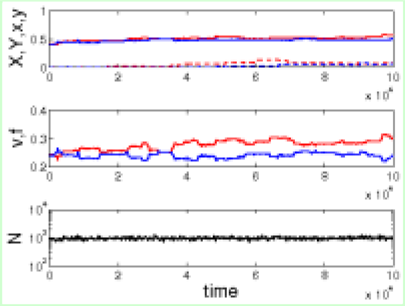

a=1.0.b=1.00.s=1.0.mu=0.00010.2.eps

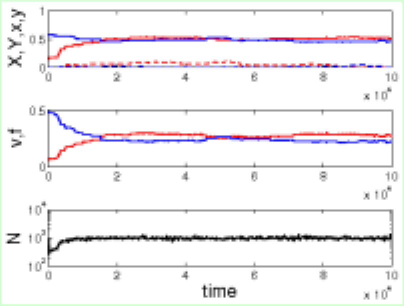

a=1.0.b=1.00.s=1.0.mu=0.00010.3.eps

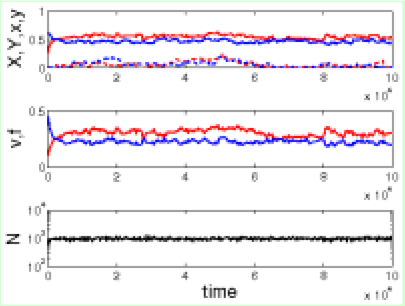

a=1.0.b=1.00.s=1.0.mu=0.00100.1.eps

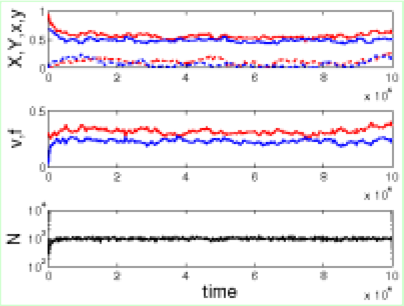

a=1.0.b=1.00.s=1.0.mu=0.00100.2.eps

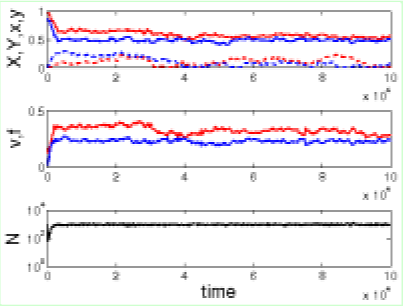

a=1.0.b=1.00.s=1.0.mu=0.00100.3.eps

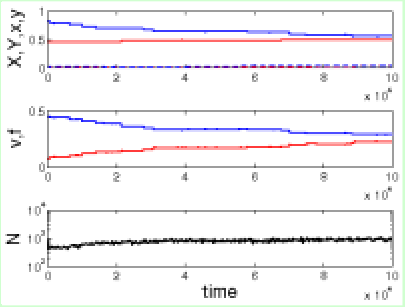

a=1.0.b=1.00.s=2.0.mu=0.00001.1.eps

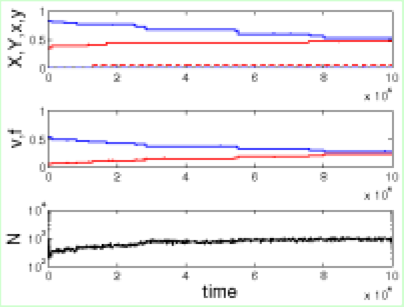

a=1.0.b=1.00.s=2.0.mu=0.00001.2.eps

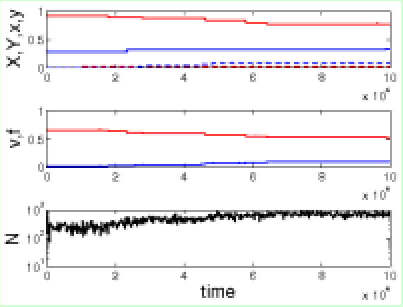

a=1.0.b=1.00.s=2.0.mu=0.00001.3.eps

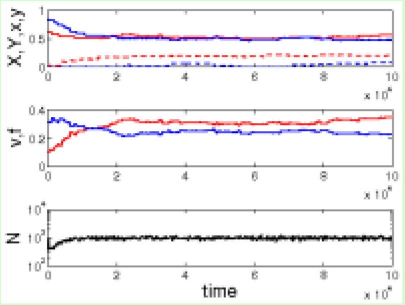

a=1.0.b=1.00.s=2.0.mu=0.00010.1.eps

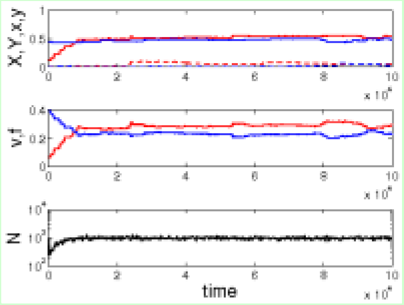

a=1.0.b=1.00.s=2.0.mu=0.00010.2.eps

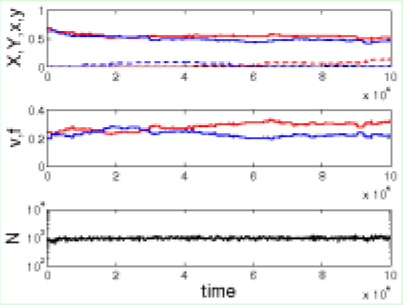

a=1.0.b=1.00.s=2.0.mu=0.00010.3.eps

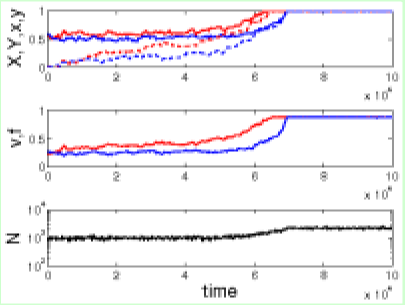

a=1.0.b=1.00.s=2.0.mu=0.00100.1.eps

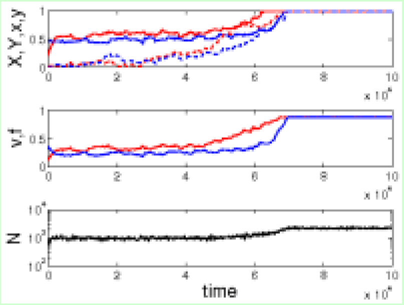

a=1.0.b=1.00.s=2.0.mu=0.00100.2.eps

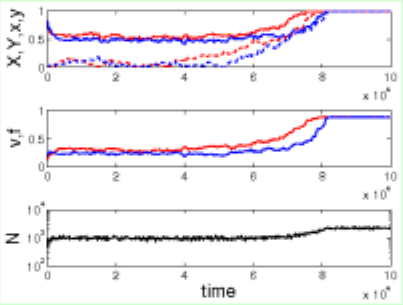

a=1.0.b=1.00.s=2.0.mu=0.00100.3.eps

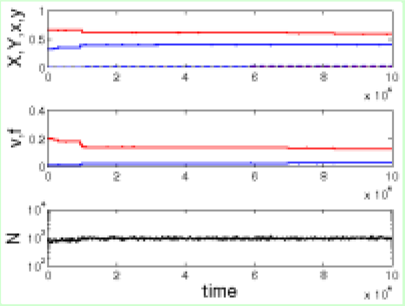

a=2.0.b=2.00.s=0.5.mu=0.00001.1.eps

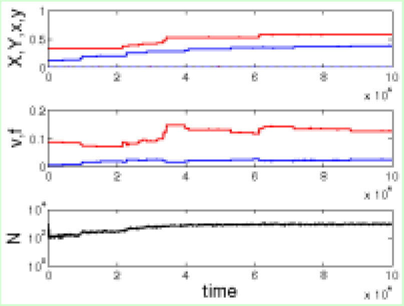

a=2.0.b=2.00.s=0.5.mu=0.00001.2.eps

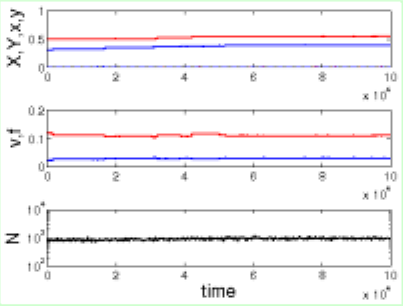

a=2.0.b=2.00.s=0.5.mu=0.00001.3.eps

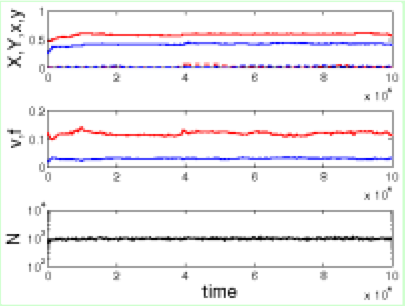

a=2.0.b=2.00.s=0.5.mu=0.00010.1.eps

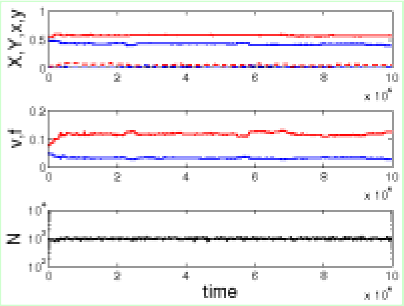

a=2.0.b=2.00.s=0.5.mu=0.00010.2.eps

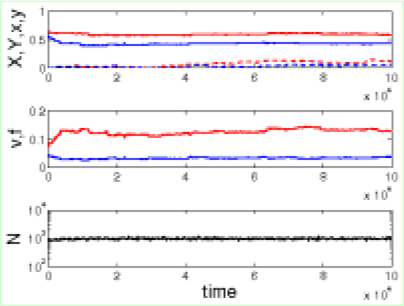

a=2.0.b=2.00.s=0.5.mu=0.00010.3.eps

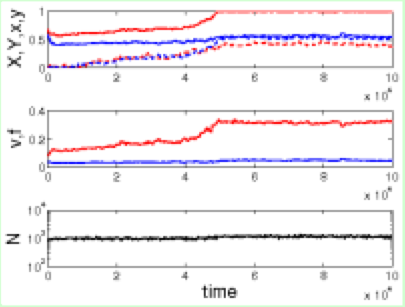

a=2.0.b=2.00.s=0.5.mu=0.00100.1.eps

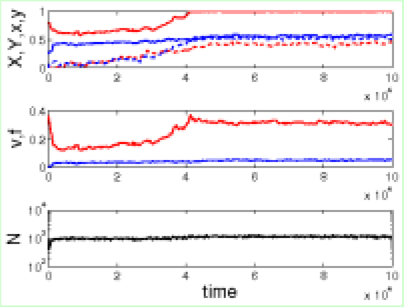

a=2.0.b=2.00.s=0.5.mu=0.00100.2.eps

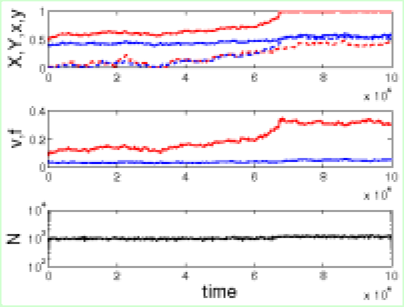

a=2.0.b=2.00.s=0.5.mu=0.00100.3.eps

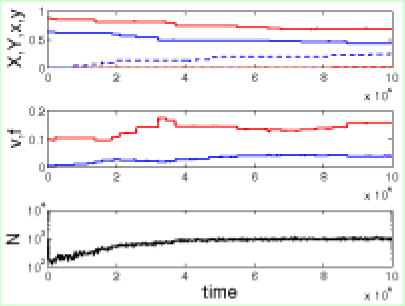

a=2.0.b=2.00.s=1.0.mu=0.00001.1.eps

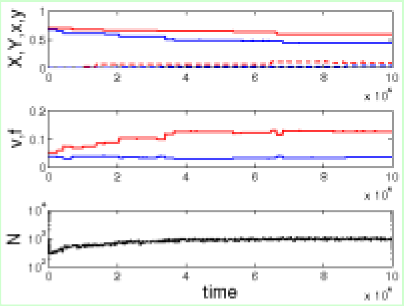

a=2.0.b=2.00.s=1.0.mu=0.00001.2.eps

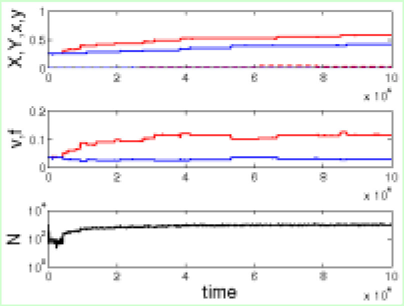

a=2.0.b=2.00.s=1.0.mu=0.00001.3.eps

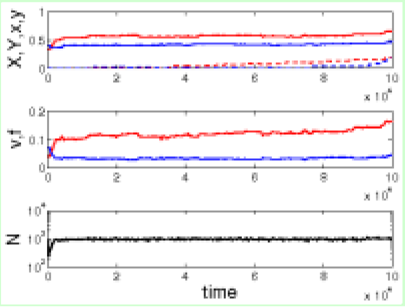

a=2.0.b=2.00.s=1.0.mu=0.00010.1.eps

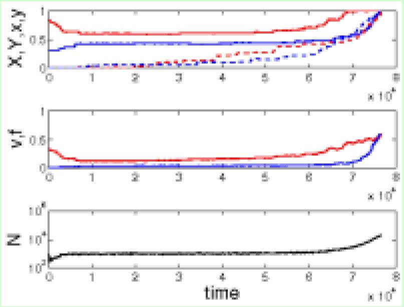

a=2.0.b=2.00.s=1.0.mu=0.00010.2.eps

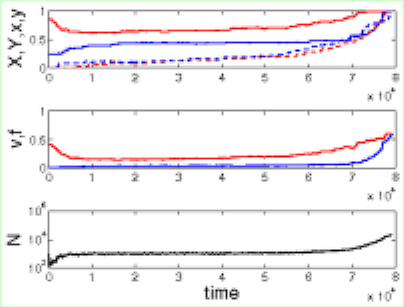

a=2.0.b=2.00.s=1.0.mu=0.00010.3.eps

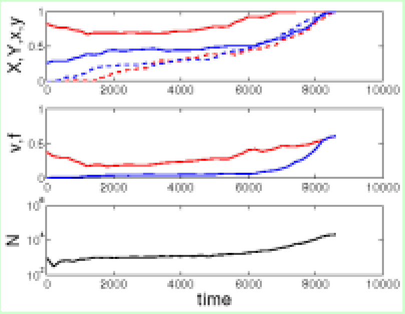

a=2.0.b=2.00.s=1.0.mu=0.00100.1.eps

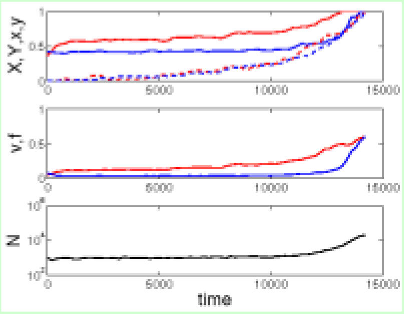

a=2.0.b=2.00.s=1.0.mu=0.00100.2.eps

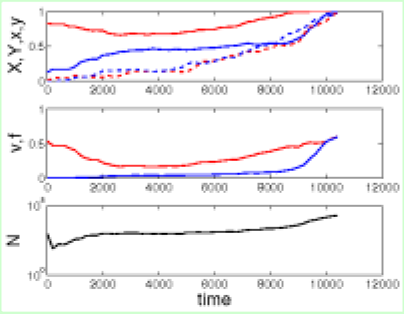

a=2.0.b=2.00.s=1.0.mu=0.00100.3.eps

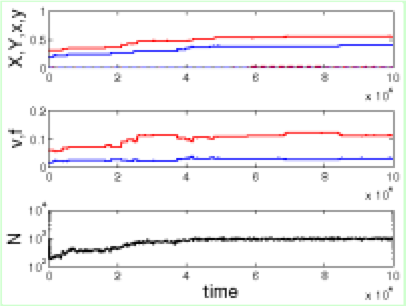

a=2.0.b=2.00.s=2.0.mu=0.00001.1.eps

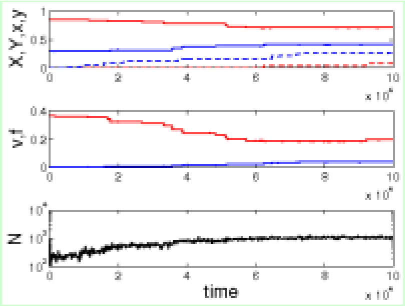

a=2.0.b=2.00.s=2.0.mu=0.00001.2.eps

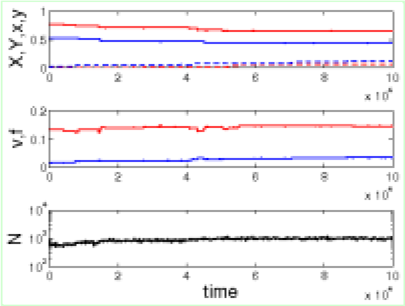

a=2.0.b=2.00.s=2.0.mu=0.00001.3.eps

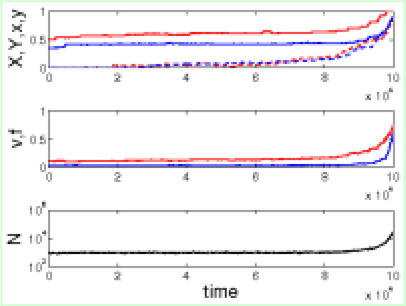

a=2.0.b=2.00.s=2.0.mu=0.00010.1.eps

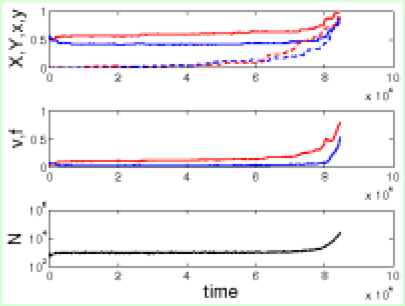

a=2.0.b=2.00.s=2.0.mu=0.00010.2.eps

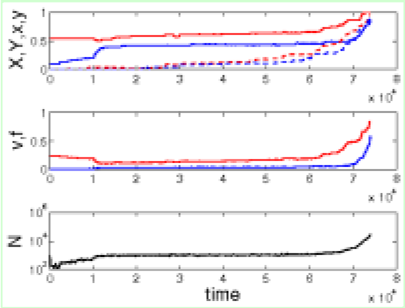

a=2.0.b=2.00.s=2.0.mu=0.00010.3.eps

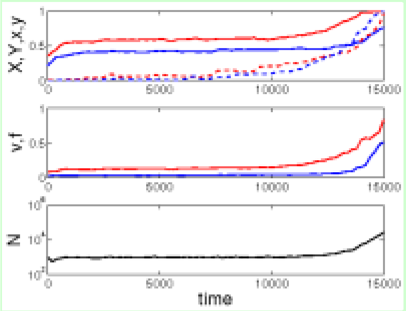

a=2.0.b=2.00.s=2.0.mu=0.00100.1.eps

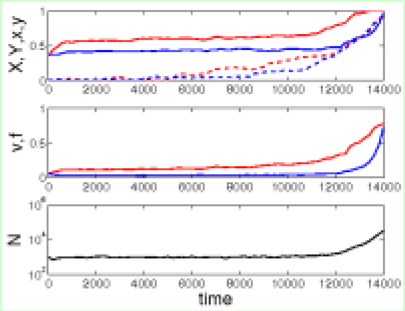

a=2.0.b=2.00.s=2.0.mu=0.00100.2.eps

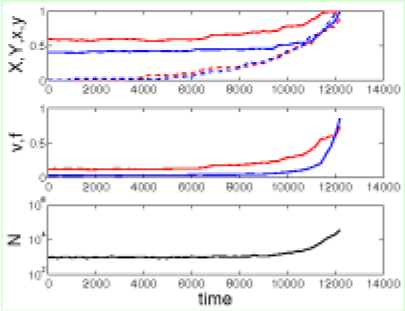

a=2.0.b=2.00.s=2.0.mu=0.00100.3.eps

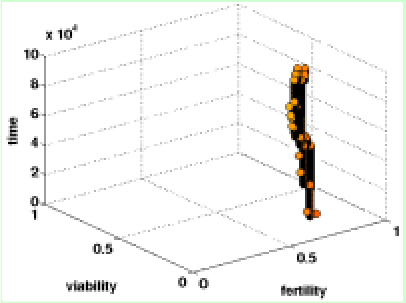

Q.a=0.5.b=0.50.s=0.5.mu=0.00001.1.eps

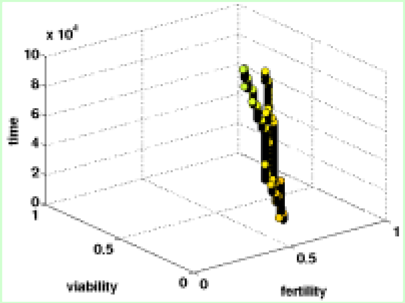

Q.a=0.5.b=0.50.s=0.5.mu=0.00001.2.eps

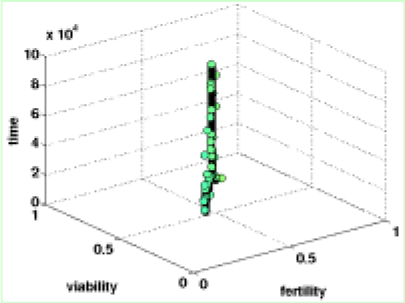

Q.a=0.5.b=0.50.s=0.5.mu=0.00001.3.eps

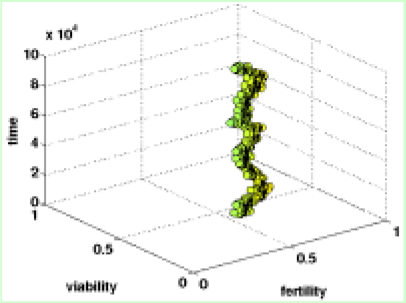

Q.a=0.5.b=0.50.s=0.5.mu=0.00010.1.eps

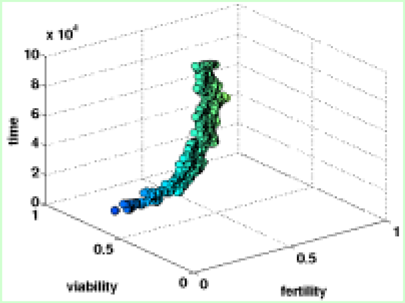

Q.a=0.5.b=0.50.s=0.5.mu=0.00010.2.eps

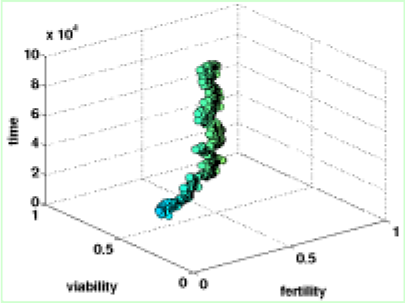

Q.a=0.5.b=0.50.s=0.5.mu=0.00010.3.eps

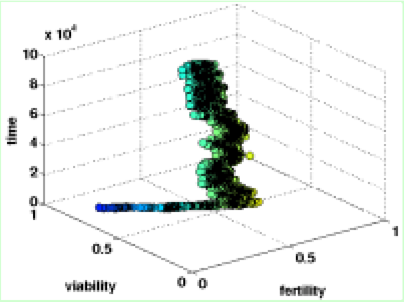

Q.a=0.5.b=0.50.s=0.5.mu=0.00100.1.eps

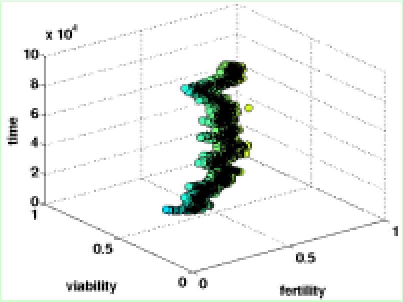

Q.a=0.5.b=0.50.s=0.5.mu=0.00100.2.eps

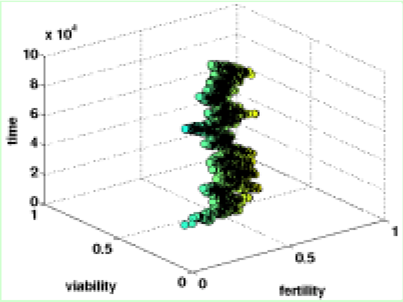

Q.a=0.5.b=0.50.s=0.5.mu=0.00100.3.eps

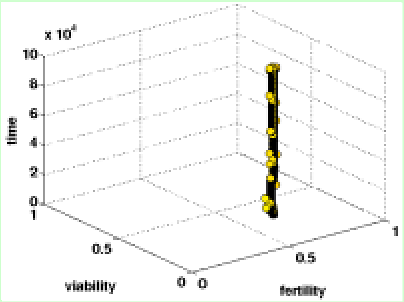

Q.a=0.5.b=0.50.s=1.0.mu=0.00001.1.eps

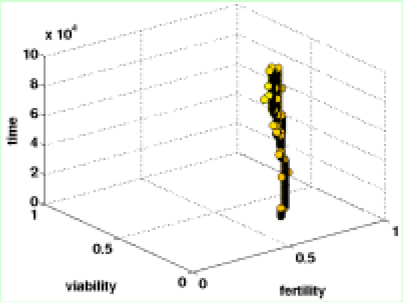

Q.a=0.5.b=0.50.s=1.0.mu=0.00001.2.eps

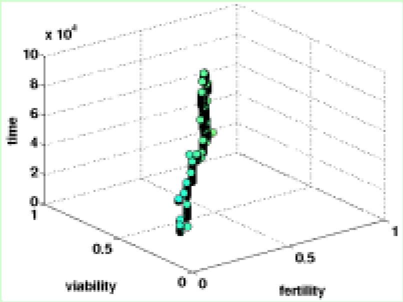

Q.a=0.5.b=0.50.s=1.0.mu=0.00001.3.eps

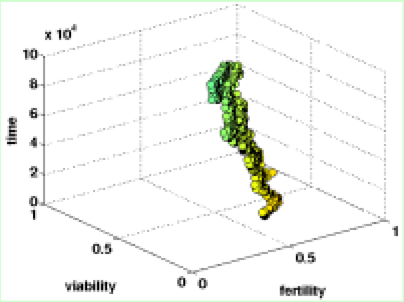

Q.a=0.5.b=0.50.s=1.0.mu=0.00010.1.eps

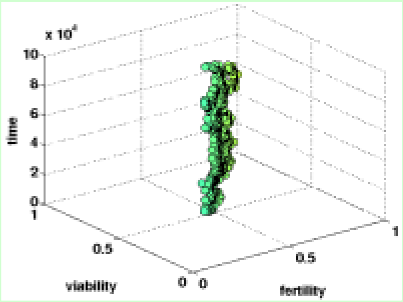

Q.a=0.5.b=0.50.s=1.0.mu=0.00010.2.eps

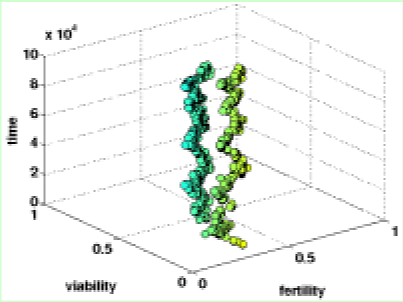

Q.a=0.5.b=0.50.s=1.0.mu=0.00010.3.eps

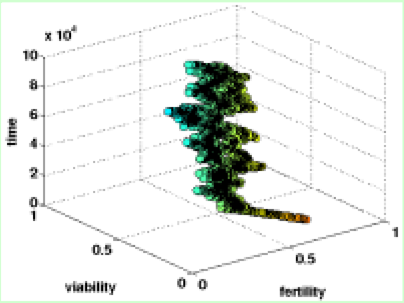

Q.a=0.5.b=0.50.s=1.0.mu=0.00100.1.eps

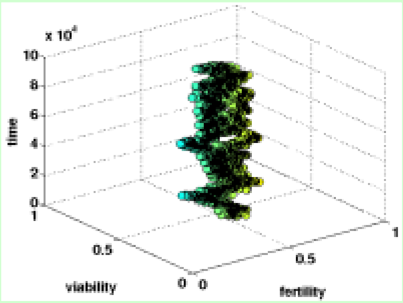

Q.a=0.5.b=0.50.s=1.0.mu=0.00100.2.eps

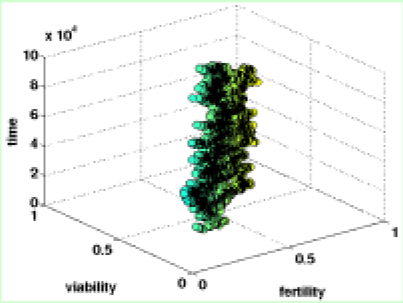

Q.a=0.5.b=0.50.s=1.0.mu=0.00100.3.eps

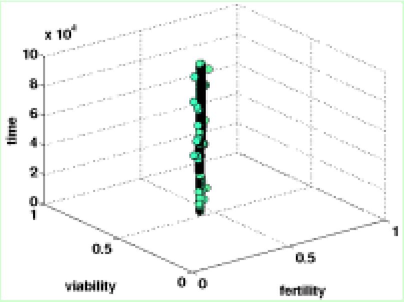

Q.a=0.5.b=0.50.s=2.0.mu=0.00001.1.eps

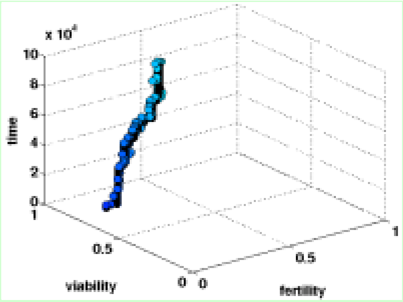

Q.a=0.5.b=0.50.s=2.0.mu=0.00001.2.eps

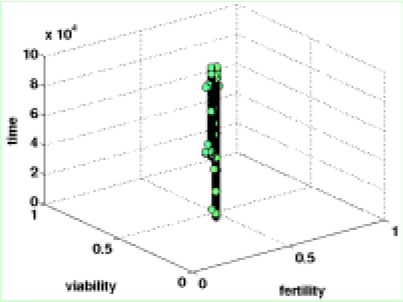

Q.a=0.5.b=0.50.s=2.0.mu=0.00001.3.eps

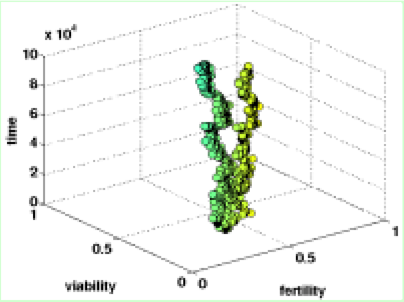

Q.a=0.5.b=0.50.s=2.0.mu=0.00010.1.eps

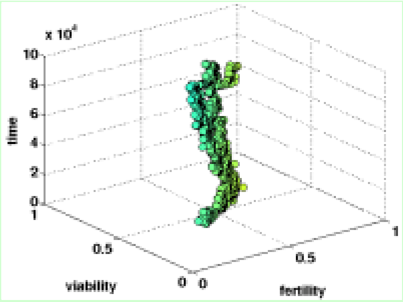

Q.a=0.5.b=0.50.s=2.0.mu=0.00010.2.eps

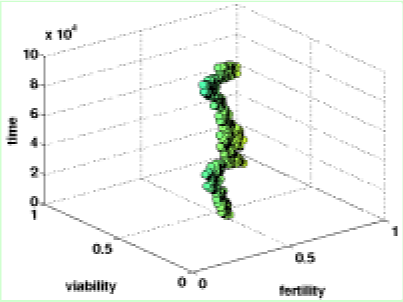

Q.a=0.5.b=0.50.s=2.0.mu=0.00010.3.eps

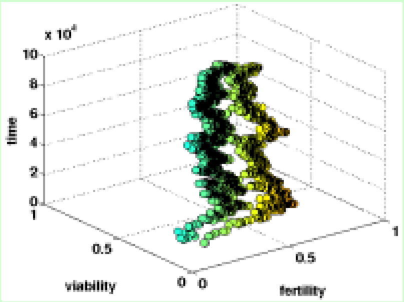

Q.a=0.5.b=0.50.s=2.0.mu=0.00100.1.eps

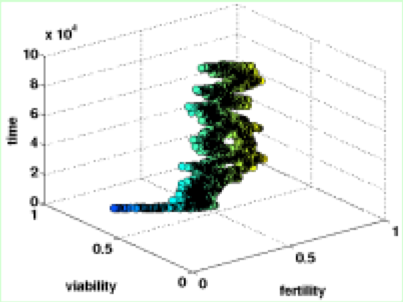

Q.a=0.5.b=0.50.s=2.0.mu=0.00100.2.eps

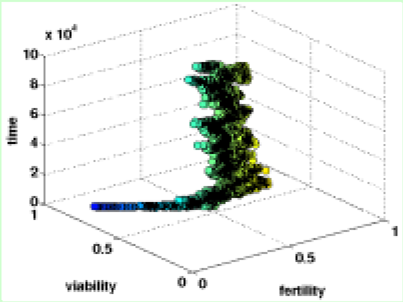

Q.a=0.5.b=0.50.s=2.0.mu=0.00100.3.eps

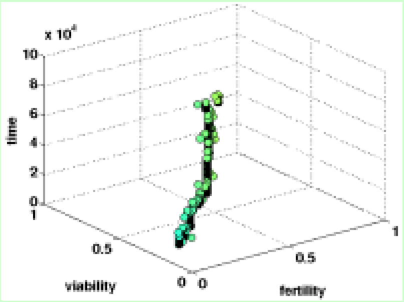

Q.a=1.0.b=1.00.s=0.5.mu=0.00001.1.eps

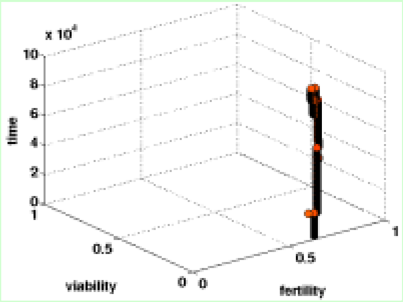

Q.a=1.0.b=1.00.s=0.5.mu=0.00001.2.eps

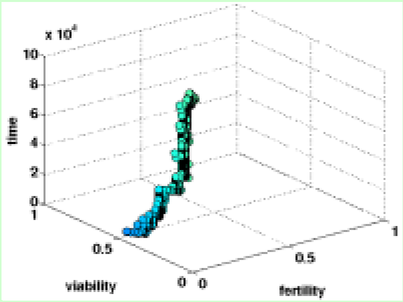

Q.a=1.0.b=1.00.s=0.5.mu=0.00001.3.eps

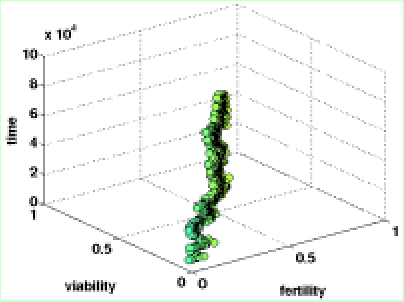

Q.a=1.0.b=1.00.s=0.5.mu=0.00010.1.eps

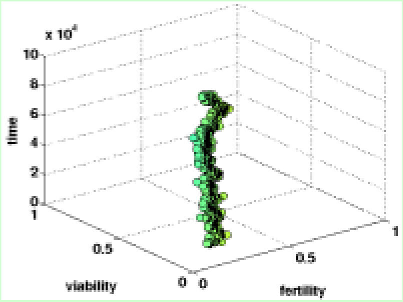

Q.a=1.0.b=1.00.s=0.5.mu=0.00010.2.eps

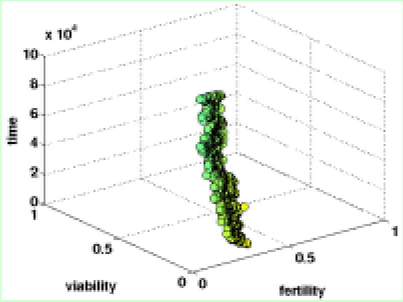

Q.a=1.0.b=1.00.s=0.5.mu=0.00010.3.eps

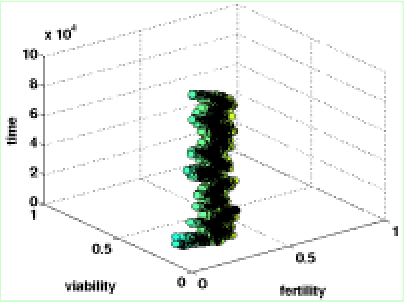

Q.a=1.0.b=1.00.s=0.5.mu=0.00100.1.eps

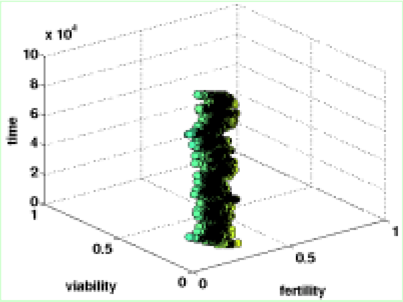

Q.a=1.0.b=1.00.s=0.5.mu=0.00100.2.eps

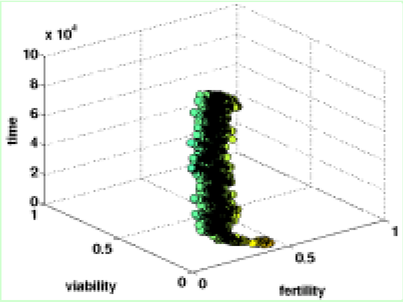

Q.a=1.0.b=1.00.s=0.5.mu=0.00100.3.eps

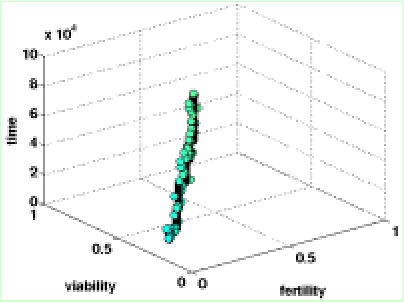

Q.a=1.0.b=1.00.s=1.0.mu=0.00001.1.eps

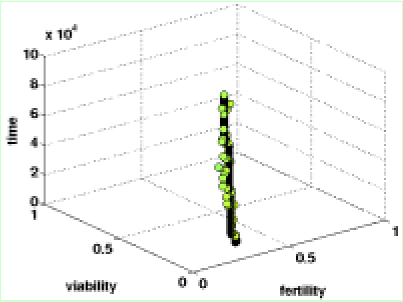

Q.a=1.0.b=1.00.s=1.0.mu=0.00001.2.eps

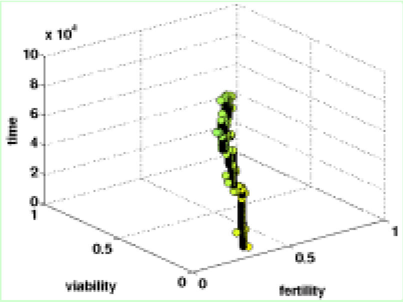

Q.a=1.0.b=1.00.s=1.0.mu=0.00001.3.eps

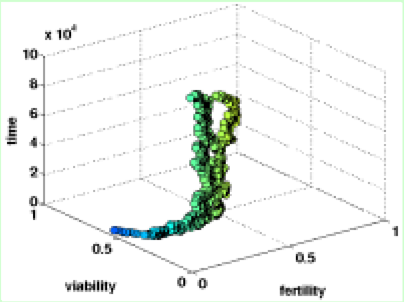

Q.a=1.0.b=1.00.s=1.0.mu=0.00010.1.eps

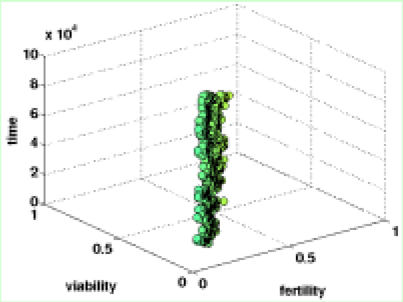

Q.a=1.0.b=1.00.s=1.0.mu=0.00010.2.eps

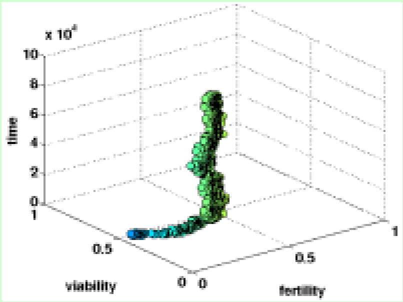

Q.a=1.0.b=1.00.s=1.0.mu=0.00010.3.eps

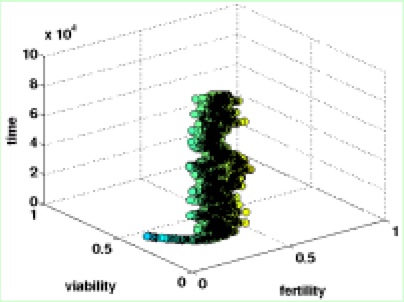

Q.a=1.0.b=1.00.s=1.0.mu=0.00100.1.eps

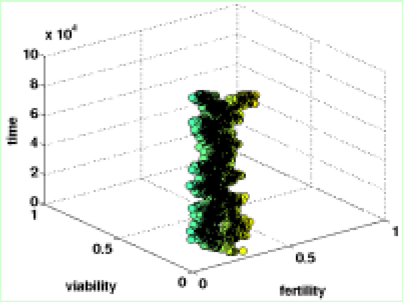

Q.a=1.0.b=1.00.s=1.0.mu=0.00100.2.eps

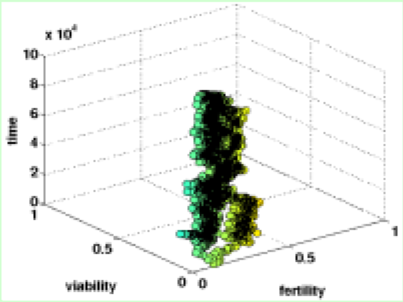

Q.a=1.0.b=1.00.s=1.0.mu=0.00100.3.eps

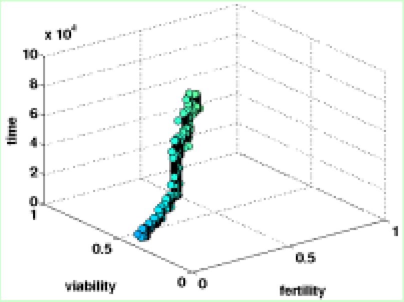

Q.a=1.0.b=1.00.s=2.0.mu=0.00001.1.eps

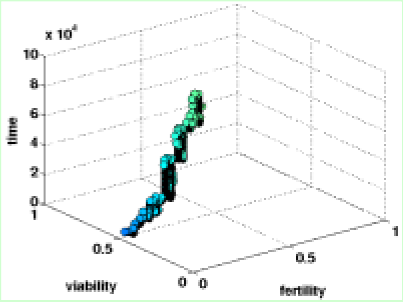

Q.a=1.0.b=1.00.s=2.0.mu=0.00001.2.eps

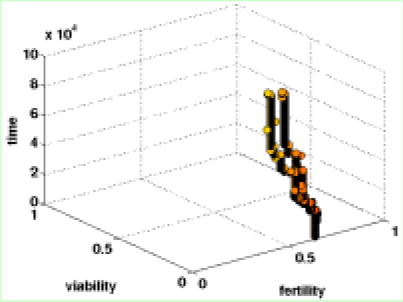

Q.a=1.0.b=1.00.s=2.0.mu=0.00001.3.eps

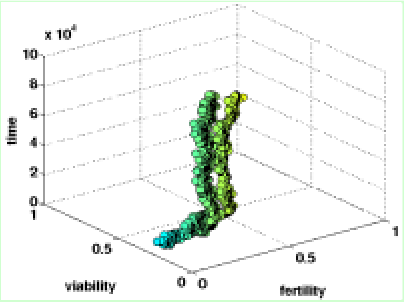

Q.a=1.0.b=1.00.s=2.0.mu=0.00010.1.eps

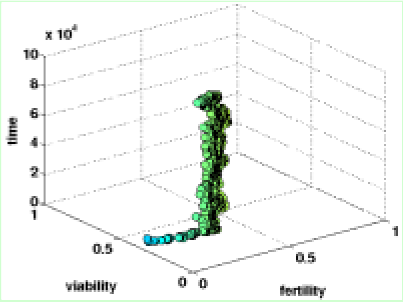

Q.a=1.0.b=1.00.s=2.0.mu=0.00010.2.eps

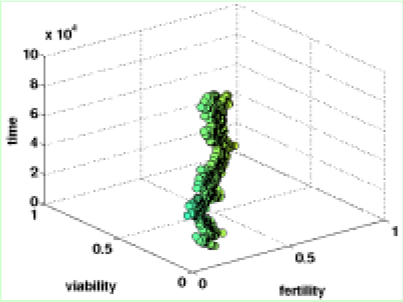

Q.a=1.0.b=1.00.s=2.0.mu=0.00010.3.eps

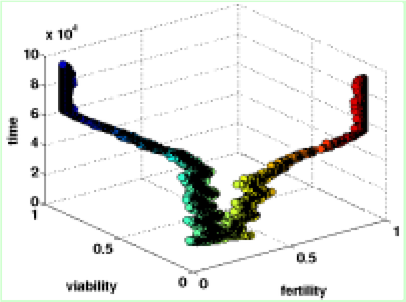

Q.a=1.0.b=1.00.s=2.0.mu=0.00100.1.eps

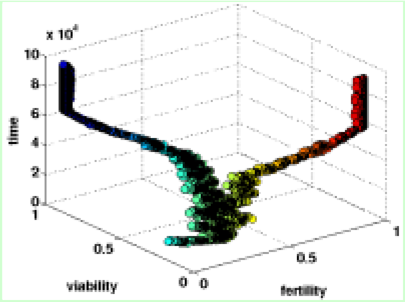

Q.a=1.0.b=1.00.s=2.0.mu=0.00100.2.eps

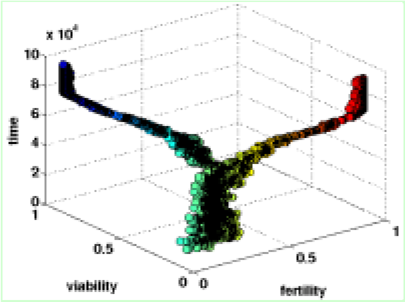

Q.a=1.0.b=1.00.s=2.0.mu=0.00100.3.eps

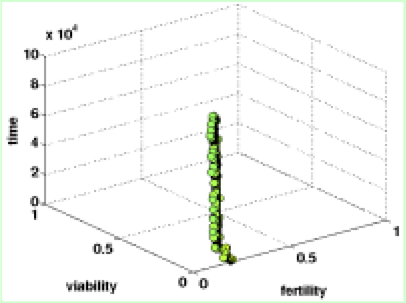

Q.a=2.0.b=2.00.s=0.5.mu=0.00001.1.eps

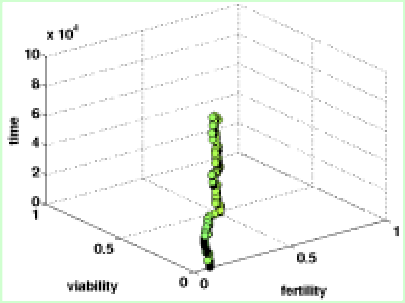

Q.a=2.0.b=2.00.s=0.5.mu=0.00001.2.eps

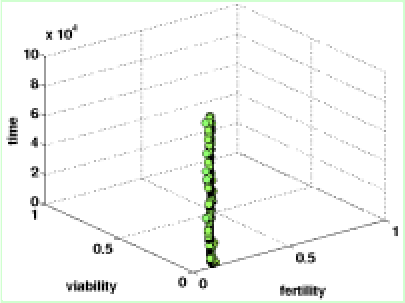

Q.a=2.0.b=2.00.s=0.5.mu=0.00001.3.eps

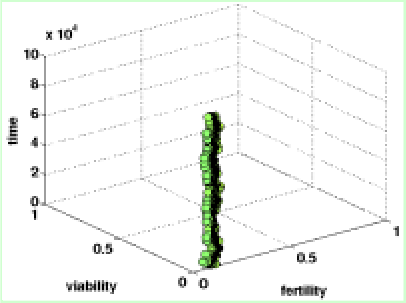

Q.a=2.0.b=2.00.s=0.5.mu=0.00010.1.eps

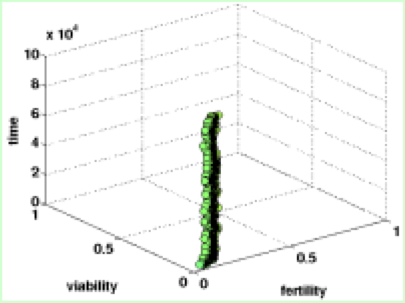

Q.a=2.0.b=2.00.s=0.5.mu=0.00010.2.eps

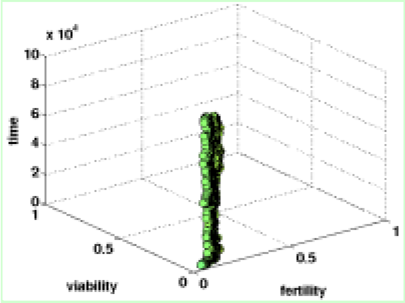

Q.a=2.0.b=2.00.s=0.5.mu=0.00010.3.eps

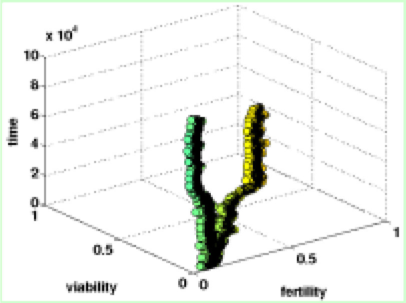

Q.a=2.0.b=2.00.s=0.5.mu=0.00100.1.eps

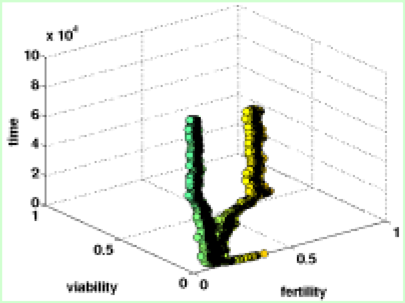

Q.a=2.0.b=2.00.s=0.5.mu=0.00100.2.eps

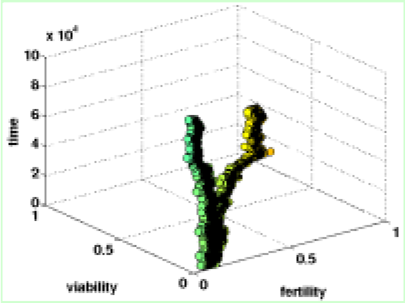

Q.a=2.0.b=2.00.s=0.5.mu=0.00100.3.eps

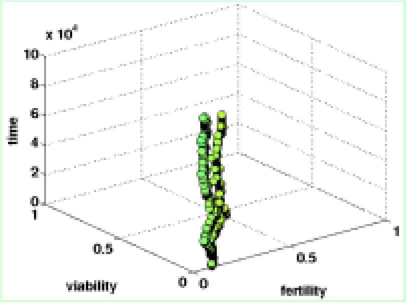

Q.a=2.0.b=2.00.s=1.0.mu=0.00001.1.eps

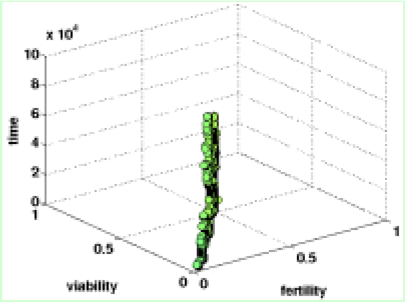

Q.a=2.0.b=2.00.s=1.0.mu=0.00001.2.DOUBLE.eps

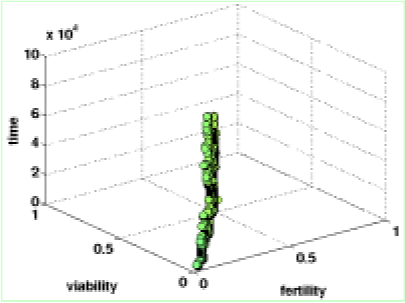

Q.a=2.0.b=2.00.s=1.0.mu=0.00001.2.eps

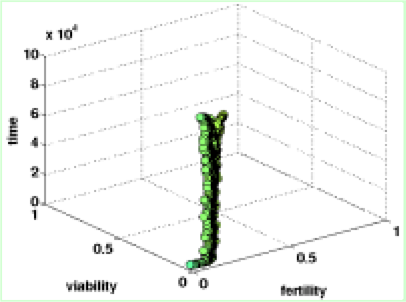

Q.a=2.0.b=2.00.s=1.0.mu=0.00010.1.eps

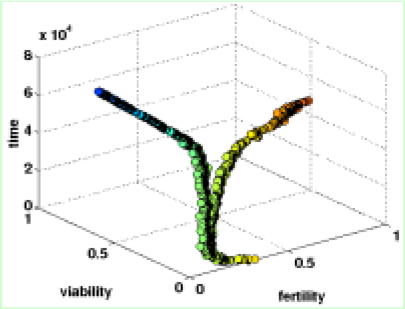

Q.a=2.0.b=2.00.s=1.0.mu=0.00010.2.eps

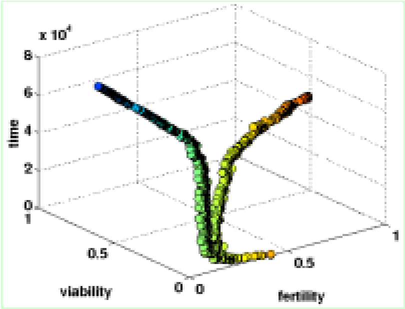

Q.a=2.0.b=2.00.s=1.0.mu=0.00010.3.eps

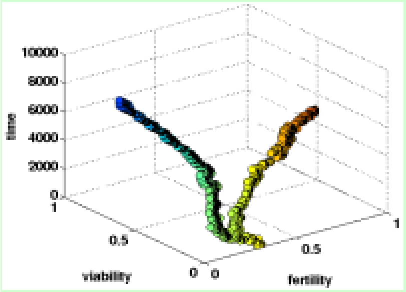

Q.a=2.0.b=2.00.s=1.0.mu=0.00100.1.eps

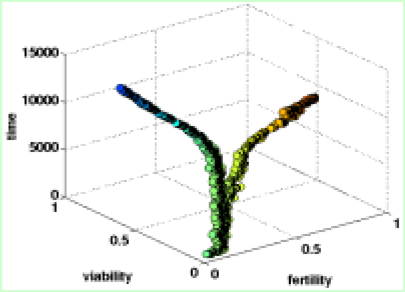

Q.a=2.0.b=2.00.s=1.0.mu=0.00100.2.eps

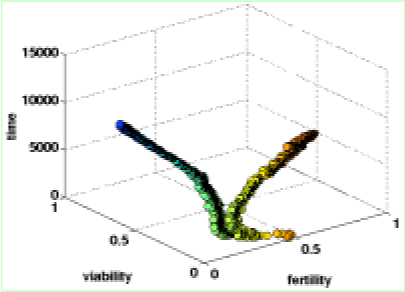

Q.a=2.0.b=2.00.s=1.0.mu=0.00100.3.eps

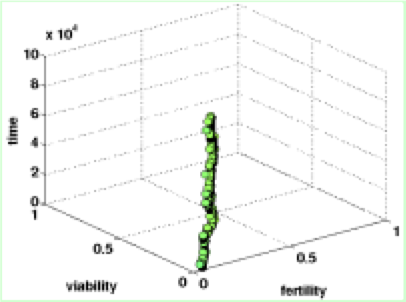

Q.a=2.0.b=2.00.s=2.0.mu=0.00001.1.eps

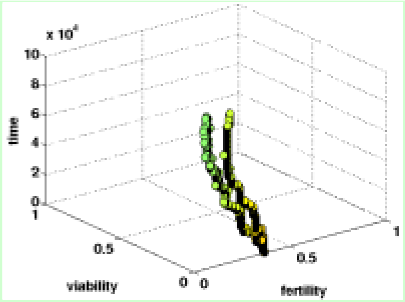

Q.a=2.0.b=2.00.s=2.0.mu=0.00001.2.eps

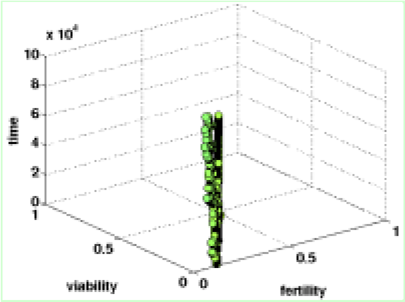

Q.a=2.0.b=2.00.s=2.0.mu=0.00001.3.eps

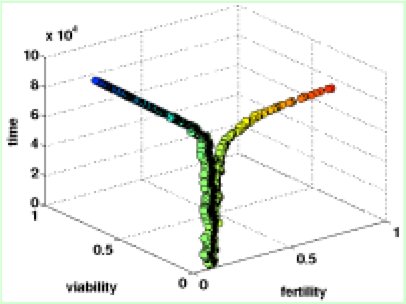

Q.a=2.0.b=2.00.s=2.0.mu=0.00010.1.eps

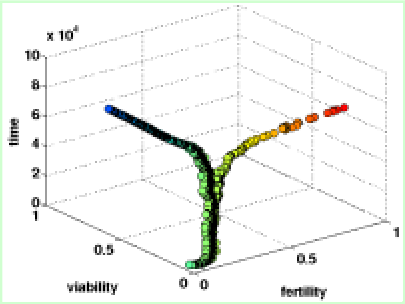

Q.a=2.0.b=2.00.s=2.0.mu=0.00010.2.eps

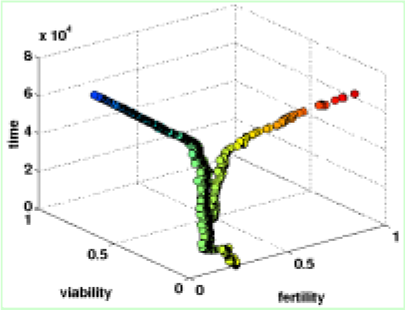

Q.a=2.0.b=2.00.s=2.0.mu=0.00010.3.eps

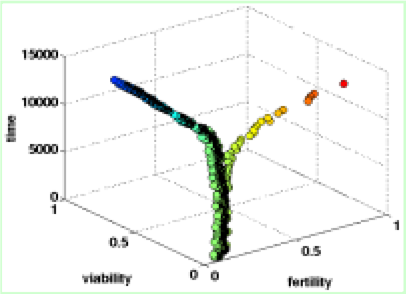

Q.a=2.0.b=2.00.s=2.0.mu=0.00100.1.eps

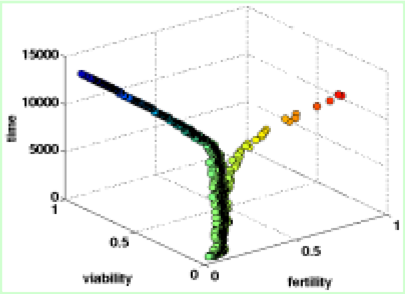

Q.a=2.0.b=2.00.s=2.0.mu=0.00100.2.eps

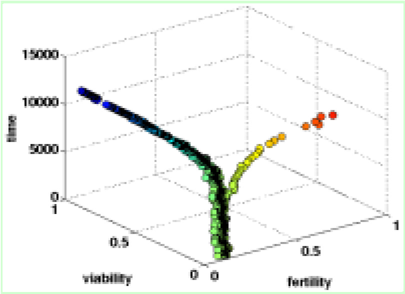

Q.a=2.0.b=2.00.s=2.0.mu=0.00100.3.eps
